# Inverse Design of Metal–Organic Polyhedra through Molecular Fragmentation and Evolutionary Optimisation

**DOI:** 10.1021/acs.jcim.5c02956

**Published:** 2026-04-01

**Authors:** Patrick W.V. Butler, Simon D. Rihm, Sebastian Mosbach, Jethro Akroyd, Markus Kraft

**Affiliations:** † Department of Chemical Engineering and Biotechnology, 2152 University of Cambridge, Philippa Fawcett Drive, Cambridge CB3 0AS, U.K.; ‡ CARES, Cambridge Centre for Advanced Research and Education in Singapore, 1 Create Way, CREATE Tower, #05-05, Singapore 138602, Singapore; § MIT, Chemical Engineering, 77 Massachusetts Avenue, Room E17-504, Cambridge, Massachusetts 02139, United States

## Abstract

Reticular materials have come to the fore of chemistry with exceptional potential in applications ranging from CO_2_ capture and chemical separations to catalysis and drug delivery. However, due to the vast combinatorial space of molecular building blocks that can form these materials, designing high-performing reticular materials for applications remains a considerable challenge. Here, we present a computational approach that combines a library of molecular fragments suitable for constructing organic building units, template-based reassembly, and evolutionary optimization to accelerate the discovery of reticular materials. Applied to metal–organic polyhedra (MOPs), this approach produces a design space of nearly 800,000 MOP configurations. A genetic algorithm (GA) based on the molecular fragments is shown to be effective at rapidly identifying optimal MOPs within this space, demonstrated through optimizing cavity properties for host–guest applications and CO_2_ interaction energies estimated by machine-learning-accelerated simulations. An important component of our approach is that it is fully ontologized and integrated within The World Avatar, forming part of a broader, interoperable knowledge model for the discovery of reticular materials.

## Graphical abstract

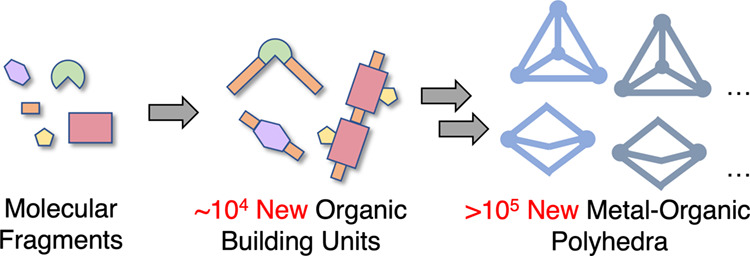

## 1 Introduction

In recent decades reticular chemistry has become established as a general approach to designing materials with targeted properties.^1–3^ In this paradigm, materials are constructed through the programmed assembly of molecular building blocks into higher-order structures with exemplary reticular materials including metal–organic frameworks (MOFs), covalent organic frameworks (COFs), and the discrete analogue of MOFs, metal–organic polyhedra (MOPs). A core attraction of reticular chemistry is the predictable assembly which enables fine control over the material properties through modifying the underlying building blocks, the potential of this being demonstrated in a diverse array of applications, including carbon capture,^4–10^ atmospheric water harvesting,^11–13^ drug delivery,^14,15^ and battery materials.^16–18^

Despite the advantages for rational design, searching for optimal reticular materials is still a considerable challenge. A primary reason for this is the expansive design space that arises from combining possible chemical building units (CBUs), which are instances of molecular building blocks, and assembly models, that define the topology and connectivity. The multitude of possible combinations precludes an entirely experimental approach even with high-throughput methods, and emphasizes the importance of developing computational methods that can rapidly explore the chemical space and return promising candidates for further study. The potential of computational methods to accelerate materials discovery is well recognized and there has been considerable focus on reticular materials, including screening known materials for applications and to gain insights into property trends^19–22^ as well as enumerating extensive libraries of hypothetical materials.^23–27^ More recently, machine learning methods have gained prominence, accelerating screening efforts and advancing inverse design through generative models capable of proposing materials or building blocks given a composition or desired properties.^28–30^ While generative models have considerable potential for interrogating structure–property relationships at scale and thereby progressing rational design, their application to reticular materials is still nascent, typically exhibiting limited explainability and requiring extensive training data sets to reliably generate valid materials. Consequently, these methods have largely been restricted to classes of materials with abundant data, particularly MOFs.^31–33^

An alternative approach for expanding the accessible chemical space of materials, which is especially applicable in cases with limited data, is to use fragmentation methods, where materials are decomposed into constituent fragments that are then recombined into new materials. The fragmentation approach has been successfully used in computational-aided design of materials, including those based on molecular cages,^34–37^ organic semiconductors,^38,39^ and molecules for singlet fission.^40,41^ The results from these studies illustrate the key benefit of the fragmentation approach: using experimentally derived fragments ensures generated structures are chemically valid (i.e., correct bonding and valency) and maximizes their synthesizability. Fragments are also advantageous in terms of optimizing material properties since they are readily encoded as genes, which when combined with a suitable fitness function, can be optimized by evolutionary algorithms.^38,39,41–50^

Previously our group applied fragmentation methods to enumerate the immediate chemical space of MOPs by combining experimentally reported CBUs and assembly models.^51,52^ As part of The World Avatar, a universal framework to connect and utilize data across domains, this data set containing 2,217 novel MOPs was integrated into a knowledge graph through the OntoMOPs ontology, which defined the relationships between CBUs, assembly models, MOPs, and properties, allowing for advanced analysis.^53–55^ Although OntoMOPs successfully captures the core concepts in MOP assembly, the focus on reported CBUs is not well-suited for incorporating hypothetical building units, and thus limits exploration of larger design spaces.

The purpose of this paper is to take the fragmentation approach for MOPs further by introducing a strategy that expands the reticular chemistry space through deconstructing reported organic CBUs into molecular fragments and reassembling them based on templates (Figure 1). We demonstrate this approach by enumerating the FragMOPs data set, comprising a total of 98,098 CBUs suitable for MOPs. The combination of these organic CBUs with four assembly models and seven metal CBUs represents a chemical space containing nearly 800,000 MOP configurations, which is captured ontologically through extensions of OntoMOPs. We further present results for optimizing MOP properties within this space through a genetic algorithm (GA) approach that is shown to effectively guide exploration toward MOPs with optimal host–guest properties. In particular, we apply the GA to optimize cavity volumes for a C_60_ guest and combine the GA with simulations accelerated by a machine learning potential in order to identify MOPs that are estimated to have strong interactions with CO_2_. This study thus provides not only a method for systematically expanding the chemical space of reticular materials in a chemically meaningful way, but further allows for explainable and effective optimization toward high-performing materials for key host–guest applications in chemical capture, catalysis, and separations.

**Figure 1:**
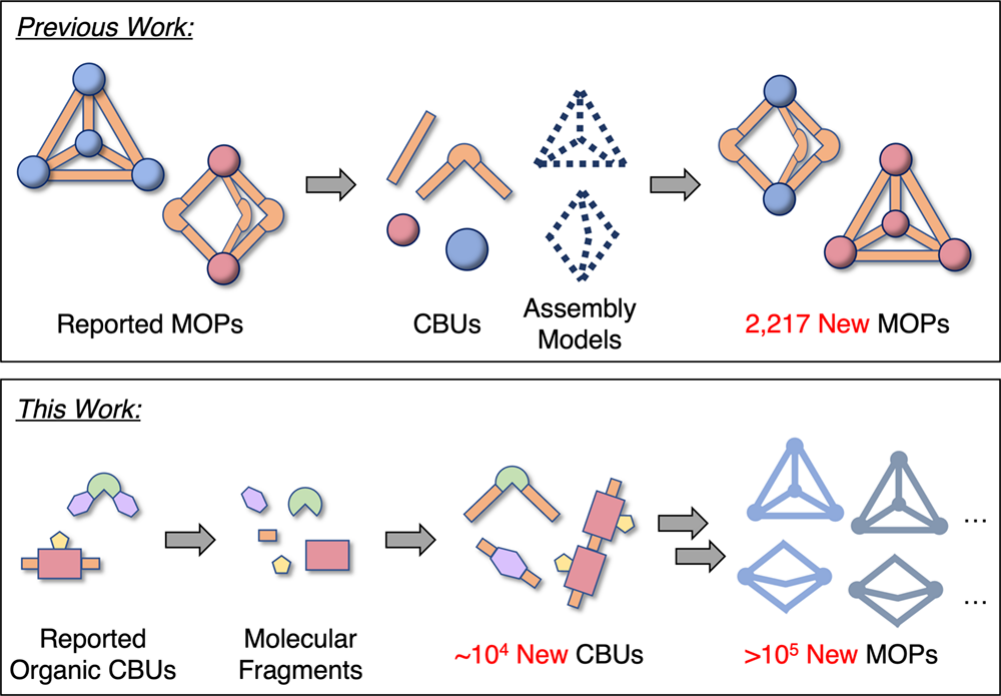
Computational approaches to exploring the MOP chemical space. Previously, we created the OntoMOPs data set by enumerating all valid combinations of experimentally reported CBUs and assembly models. By contrast, in the FragMOPs approach described here reported organic CBUs are decomposed into molecular fragments and then recombined into new CBUs that can be assembled into orders of magnitude more MOPs using the same metal units and assembly models.

## 2 Methods

### 2.1 Molecular Fragmentation

The initial library of organic CBUs was compiled by merging those from the previously reported OntoMOPs data set with the HEALED data set,^56^ which contains CBUs derived from experimental MOF structures. Each of the collected CBUs was then processed to fragments by cleaving all exocyclic, non-hydrogen single bonds and replacing each fragmentation point with dummy atoms at either end to denote available bonding sites. This approach was selected to favor cross-coupling sites, however, it is important to note that different fragmentation approaches, such as BRICS,^57^ could yield significantly different fragments and design spaces. To allow for subsequent, controlled functionalization, fragmentation points that resulted in fragments with one bonding site, corresponding to a side chain group, were replaced by a hydrogen atom on the parent fragment. Following this, the unique fragments were collected based on canonical SMILES and were categorized according to the number of bonding sites: fragments with one bonding site were marked as side chain fragments, those with two as linker fragments, and those with three or more as node fragments. An additional category for binding group fragments was manually defined based on the carboxylate and pyrazolate binding groups in the OntoMOPs data set, with the defined fragments excluded from the categorization. All final fragments were instantiated in the knowledge graph and linked to data detailing their type, SMILES, extracted geometry, and chemical properties (see Figure S.2).

### 2.2 CBU Assembler

Given a CBU template and a set of fragments containing at least a binding group fragment and optionally linker and node fragments, the CBU assembler first checks that each position in the template is satisfied and, if so, then orders the linker fragments based on the designated sequence in the template (Figure 2). The fragments are then joined starting from the binding group, appending the linker fragments in sequence, and then either appending a second binding group at the end, or if a node fragment is provided, appending copies of the partially assembled structure to each bonding site on the node fragment. The orientations of linker fragments are controlled by setting which bonding site will be connected first, represented as a vector with values of 0 or 1 for each linker. For rapidly enumerating the space SMILES-based assembly is supported. When a geometry is required, an alternative protocol is invoked that operates on the geometries recorded with each fragment. In this geometry-based assembly, a local coordinate system is determined for the current fragment using the selected bonding site dummy atom and the two nearest neighboring atoms. A transformation is then calculated to align this coordinate system with a similarly defined local system on a second fragment (or partially assembled structure) at an appropriate separation and orientation. Effectively, this results in mapping the dummy atom of one bonding site onto the neighboring atom of the other and *vice versa*. The bonding site dummy atoms are then removed and a new single bond is created between the atoms neighboring the bonding sites of each fragment. At this point, a distance matrix is calculated to check for overlaps between nonbonding atoms. If so, the torsion angle around the new bond is incremented iteratively until either the overlaps are eliminated or the angle exceeds 360 deg, in which case the assembly is failed. Alternatively, through interfacing with RDKit, an option is provided to energy minimize the assembled CBU geometry using the MMFF94 force field.^58^ Finally, to make the CBUs able to function as intended, the binding groups are aligned, either to each other in the case of 2-linear CBU templates, or otherwise to a plane that contains the center point of each binding group. For enumerating CBU combinations from a list of fragments and a CBU template, the combinatorial product of the sets of all fragments accepted at each position is determined. By default, if asymmetric linkers are present all unique orientations are attempted for each combination. Alternatively, if the symmetric constraint is enabled, the orientations of asymmetric linkers with more than one position are constrained to alternate. To generate MOPs from the created organic CBUs we use our MOP assembler reported previously, which is a purely geometric approach that positions the building units according to an assembly model.^52,54^

**Figure 2:**
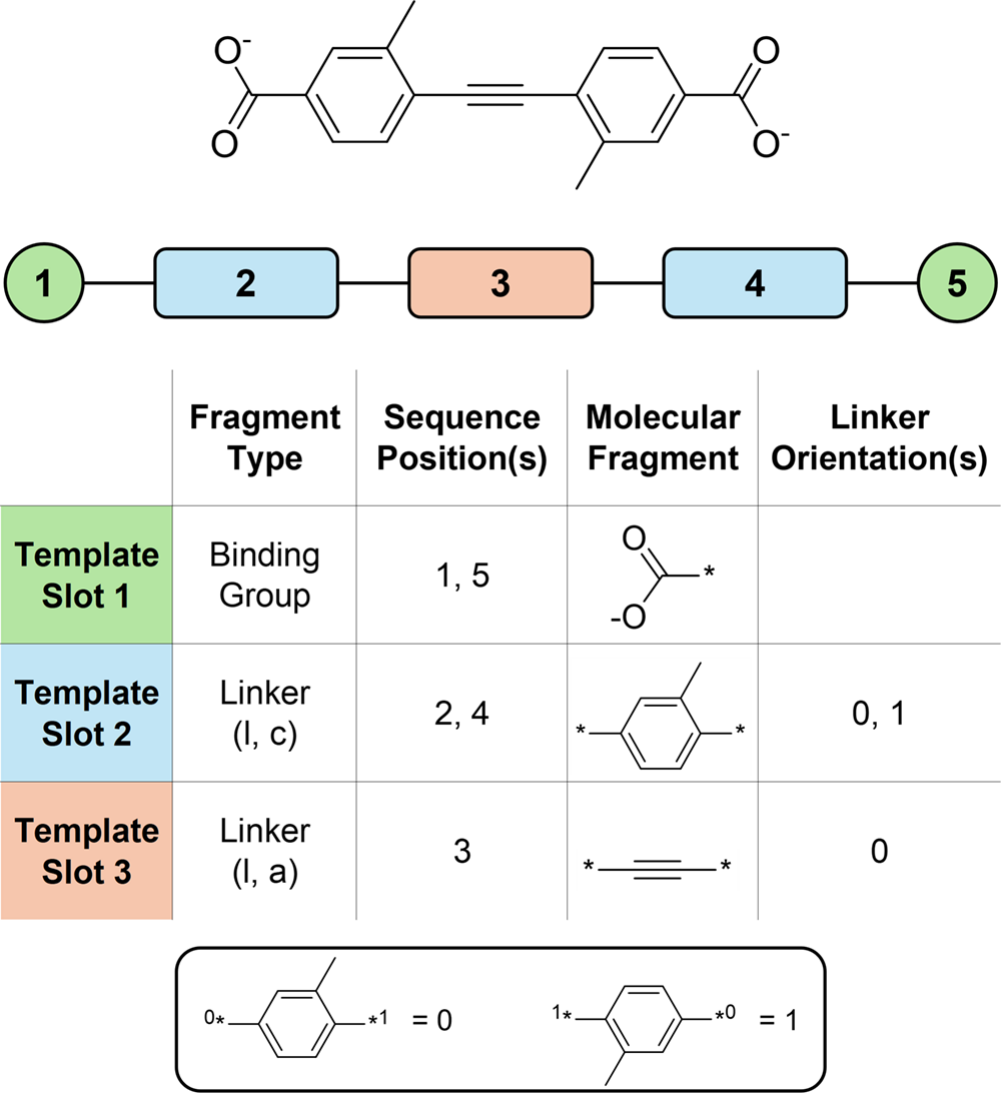
Overview of the relationship between a CBU and its underlying template, shown for a representative example. The template consists of fragment slots that specify both fragment type and sequence position. The position guides how fragments are connected together to form the complete CBU, with linker fragments assigned explicit positions, whereas binding group and node fragments occupy the terminal sequence positions. To realize a CBU requires providing an appropriate fragment for each slot and, in the case of linkers, setting the orientation (0 or 1). An insert below illustrates the linker orientations. Legend: l = linear, c = cyclic, a = acyclic, ^★^ = bonding site.

### 2.3 Genetic Algorithm

To efficiently explore and optimize within the FragMOPs design space, we developed a GA adapted to describe the MOP components. In the implementation, each candidate MOP is represented by a chromosome, consisting of genes encoding its assembly model, metal CBU, the molecular fragments occupying each position of the organic CBU template, and, where relevant, the orientations of linker fragments in the template. This chromosome is defined in practice as a sequence of values (i.e. a vector), where, for example, the value at the first position selects the assembly model from a predefined list. The CBU template is not included in the chromosome. Consequently, the lists of assembly models, fragments, and metal CBUs needs to be filtered for each template beforehand, and the length of the chromosome varies depending on the number of fragment slots the CBU template contains. Nevertheless, with the relatively small number of templates, it is simpler and more efficient to parallelize over the templates. This also greatly reduces dimensionality, thereby improving convergence, and allows exploring CBU topologies independently.

To begin the optimization an initial population of chromosomes is generated randomly. Thereafter, at each generation, the ranking of individuals is determined using a defined fitness function, which incorporates properties calculated for the structure returned by the MOP assembler. Our implementation includes elitism so that the top ranked individuals are copied to the next generation. The remainder of the population is created through tournament selection, gene crossover, and mutation, with an option allowing for duplicate chromosomes to be replaced with random ones. In the optimizations reported we use a population of 30 individuals evolved for 30 generations. The mutation probability was set at 0.15, the crossover probability at 0.9, and the elitism proportion was 0.05, with all parameters remaining constant throughout. Further details for each optimization are available in the supplementary files.

### 2.4 Knowledge Graph and The World Avatar Integration

FragMOPs extends the framework established in OntoMOPs by fully encoding the concepts and relationships between fragments, CBUs, CBU templates, and MOPs within a semantic knowledge graph. This structured representation enables automated reasoning for data validation, constraint enforcement, and complex querying. For example, within the CBU templates, encoded relationships define which fragment types are valid at each position and impose constraints such as atom count, cyclicity, and linearity.

In line with our previous work on reticular materials, FragMOPs is implemented as part of The World Avatar (TWA), a dynamic ecosystem of interconnected data and knowledge models built on FAIR principles.^59^ This integration links FragMOPs to other chemical knowledge bases, including OntoSpecies,^60^ an ontology describing chemical species. Moreover, TWA hosts a diverse suite of knowledge models, such as OntoCompChem^61^ and OntoPESScan,^62^ creating future opportunities to interoperate with complementary domains of chemistry knowledge and beyond. Through this integration, FragMOPs contributes to our ongoing work within The World Avatar to develop a holistic knowledge model for reticular materials discovery.^53–55^

## 3 Results

### 3.1 FragMOPs Chemical Space

Fragmentation of the organic CBUs from the combined data set produced 71 unique fragments following manual curation to prioritize simple and general fragments. This set comprises two binding group fragments (carboxylate and pyrazolate), two node fragments with three bonding sites, five nonlinear noncyclic linkers, 24 side-chain fragments, 20 linear cyclic linkers, and 18 linear noncyclic linkers. A further 24 asymmetric linear linker fragments were created through performing single C–H substitutions on a benzene linear linker with each of the 24 side-chain fragments.

With this fragment library in hand, a series of complementary CBU templates were defined based on 2-linear, 2-bent, and 3-planar CBUs in the OntoMOPs data set, where the notation refers to the number and relative arrangement of the binding site vectors (see Figure S.1). In total eight CBU templates were defined: four 2-linear templates, two 2-bent, and two 3-planar (see Table S.1). These were selected as representative examples that capture common topologies observed in MOPs, providing a set that is diverse yet amenable to detailed analysis for demonstrating the FragMOPs approach. In all cases the templates were defined to accept a single binding fragment such that CBUs with mixed binding groups would not be generated.

To assess the chemical space enclosed by these fragments and templates, we then enumerated all valid CBUs using the SMILES assembly method with all linker orientations allowed. In total this returned 98,098 unique SMILES and was completed in just 138 s on a single CPU core. Compared to the previous OntoMOPs data set that contained 101 organic CBUs, 19 of which align with the defined CBU templates, this represents a 971-fold increase in available CBUs and a 5,163-fold greater sampling of the mutual design space. The considerable expansion in the chemical space is illustrated by a t-SNE projection of Morgan fingerprints calculated for the previous CBUs and the FragMOPs CBUs (Figure 3A), with the two hemispheres observed in the plot found to correspond to the two binding groups (Figure S.4).

**Figure 3:**
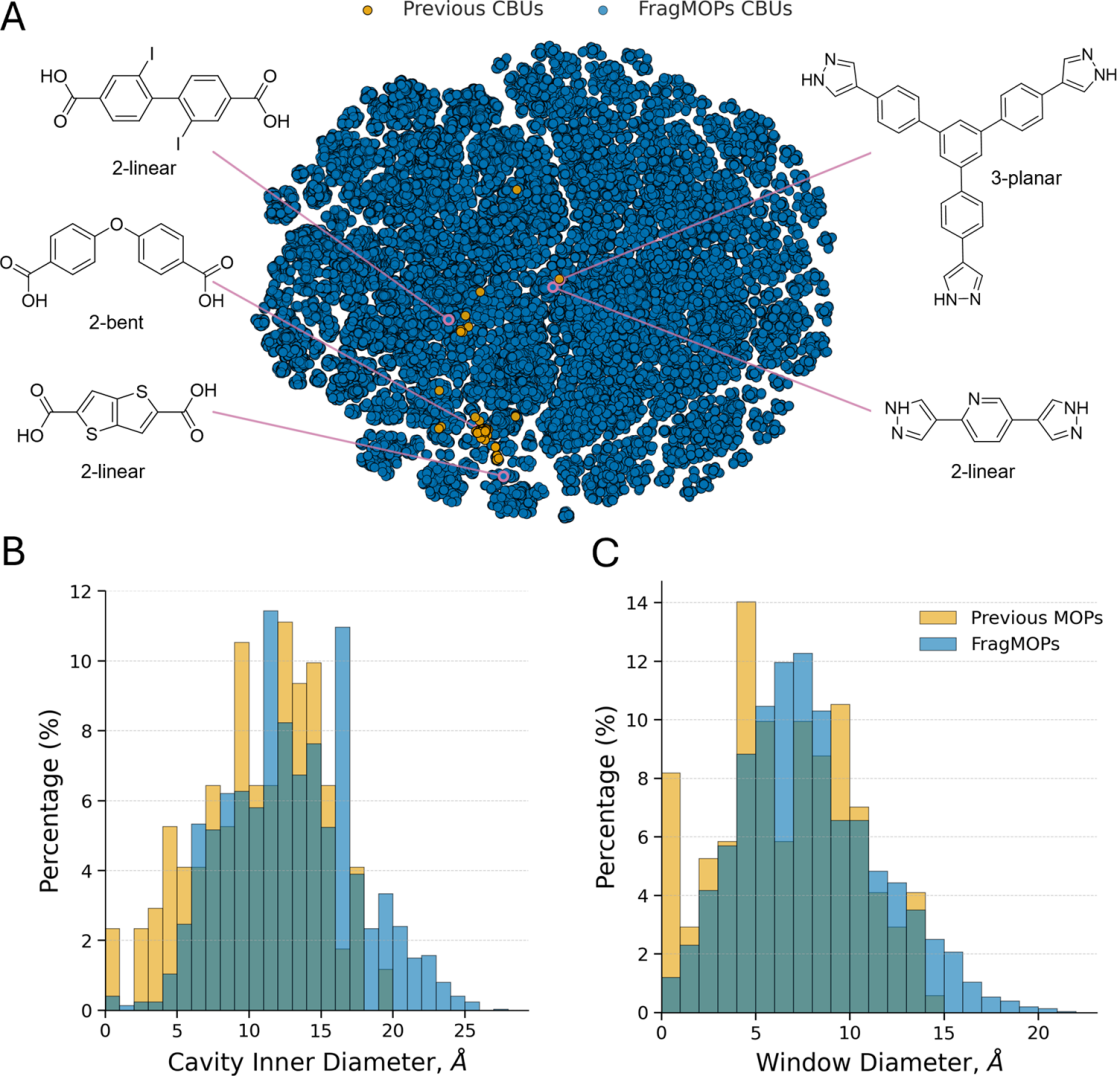
Expansion and characterization of the FragMOPs chemical space. A t-SNE embedding of 2048-bit Morgan fingerprints depicts the chemical space of organic CBUs from the previous OntoMOPs data set and the newly generated FragMOPs CBUs (A) annotated with examples of commercially available CBUs present only in the FragMOPs data set and their type. Distributions of inner-sphere (B) and window (C) diameter for 202 MOPs from the previous data set and 3000 randomly sampled FragMOPs with mutual assembly models illustrates the broader and more continuous range of properties in the FragMOPs data set.

To further characterize this expanded chemical space, we computed several molecular property distributions. The distribution of molecular weight for the FragMOPs CBUs is found to be shifted significantly to a higher mass average of 615.21 g mol^–1^ compared to the previous organic CBUs, which had an average of 332.73 g mol^–1^ (Figure S.5). While this is a significant increase, it is largely an expected consequence from the combinatorial increase in CBUs containing two to three linker fragments, which are relatively less common in the previous data set. Additionally, the FragMOPs data set contains heavy fragments, such as iodide, that are not present in the OntoMOPs data set.

We next evaluated synthetic accessibility using the SAscore metric.^63^ For the FragMOPs CBUs we find that the mean SAscore is higher than that for the previous organic CBUs, 3.06 to 2.36 (Figure S.6). Interestingly, the maximum SAscore is lower for FragMOPs (5.86 vs 6.50), with the higher value for the previous set of CBUs arising from pyrogallolarene- and metal-containing species outside the FragMOPs design space. Excluding these, the maximum drops to 3.07. Given that a SAscore of around 3 corresponds to the average for commercially available compounds,^63^ this analysis indicates that the FragMOPs CBUs are broadly synthetically accessible. Indeed, a search of the ZINC in-stock database^64^ identified 100 commercially available CBUs, of which only 16 are present in the previous OntoMOPs data set (full list detailed in Section S.5.3).

For exploring the diversity of MOPs accessible through FragMOPs, we next implemented a workflow using the geometry-based protocol of the CBU assembler without energy optimization to generate geometries which could be input to the MOP assembler. The other inputs required for the MOP assembler are an assembly model and a metal CBU. For the assembly models, we selected three assembly models that are commonly reported experimentally with the CBU templates and feature varying stoichiometries: (3-pyramidal)_2_(2-bent)_3_, (3-pyramidal)_4_(2-linear)_6_, and (3-pyramidal)_4_(3-planar)_4_. Metal CBUs for the 3-pyramidal sites, which form the vertices in these assembly models, were extracted from the OntoMOPs data set, yielding seven CBUs. Under these conditions, the total number of possible MOP configurations is estimated to be 791,056. In comparison to the 202 MOPs from the previous OntoMOPs data set within the same chemical space, this represents increasing the design space by in excess of three orders of magnitude. From this multitude of configurations, we assembled a subset of 1,000 MOPs per assembly model (3,000 total) using randomly selected metal and organic CBUs. For each MOP we calculated the cavity properties including the maximum inner sphere diameter and window diameter to compare with the 202 MOPs from the previous data set. These results indicate that, despite differences in the underlying chemical spaces, the shared CBU templates yield distributions with comparable overall statistics. The average inner-sphere diameters are 10.5 Å for the previous data set and 12.9 Å for the FragMOPs data set, while the average window diameters are 6.6 Å and 7.9 Å, respectively. The FragMOPs data set exhibits larger maximum inner-sphere and window diameters (27.8 Å and 21.2 Å) compared to those of the previous data set (19.3 Å and 14.3 Å), reflecting the inclusion of larger fragments derived from the HEALED data set. Although the overall statistics are similar, the more continuous coverage observed in the FragMOPs distributions highlights its enhanced tunability of cavity properties across the assembly models.

### 3.2 MOP Optimization

Having created a large and scalable design space for MOPs, we next sought to explore optimizing within the space to identify promising candidates for applications. The fragment-based approach lends itself naturally to optimization with a genetic algorithm, and therefore to assess the potential of such an approach, we examined two applications: optimizing MOP pore volumes for a C_60_ guest and optimizing average CO_2_ interaction energies.

#### 3.2.1 Cavity Structure

For host–guest applications a crucial factor is the complementarity between the shape of the guest and the cavity of the host. In the case of C_60_, we thus chose to optimize the spherical volume of the cavity to be 1.05× the van der Waals volume of C_60_, which resulted in a target volume of 625 Å^3^. While we do not expect this is a complete descriptor of host performance, it does represent an inexpensive target to demonstrate optimizing the MOP cavity properties from the CBU fragments. To favor synthetically accessible and structurally simple candidates, the fitness function combined three weighted terms: deviation from the target cavity volume, SAscore, and the number of side chain fragments. The weights were fixed with a relative ratio of 1:10:10. In cases where the only information is the atom connectivity, determining the last term would require significant processing similar to the fragmentation protocol used initially. However, with the FragMOPs knowledge graph, this can be simply queried from the CBU.

With the fitness function determined, we then set the search space to consist of the three assembly models from above, (3-pyramidal)_2_(2-bent)_3_, (3-pyramidal)_4_(2-linear)_6_, and (3-pyramidal)_4_(3-planar)_4_, and three representative metal CBUs, [V_3_O_2_(OH)_2_(HCO_2_)_3_], [Zr_3_O­(OH)_3_(C_5_H_5_)_3_], and [V_6_O_6_(OCH_3_)_9_(SO_4_)], corresponding approximately to small, medium, and large sized CBUs. The organic CBU space was restricted by enforcing that the CBUs be (pseudo)­symmetric to maintain equivalent coordination environments, which is desirable synthetically to avoid forming mixtures of isomers. Overall, with these settings the search space contains an estimated 51,100 organic CBUs and a total of 153,300 MOP configurations.

The optimization to the target inner sphere volume within the defined search space aggregated across assembly models is shown in Figure 4. These show that high-fitness candidates with cavity volumes close to the target are generated even within the random initialization, and convergence to optimal solutions is achieved in only a few generations. The fittest MOPs achieve the desired characteristics in terms of cavity size and synthetic accessibility, with inner sphere volumes between 614 and 621 Å^3^ and SAscore values between 1.57 and 2.59. The population averages also rapidly converge to the target volume, the (3-pyramidal)_4_(2-linear)_6_ and (3-pyramidal)_4_(3-planar)_4_ converging in around 10 generations while the (3-pyramidal)_2_(2-bent)_3_ model takes slightly longer at approximately 15 generations. Analyzing the individual runs (Figure S.14) we expect this is due to template 1 for the 2-bent CBU only having relatively few configurations that reach the target volume, and hence any mutations or crossover with other genes leads to lower fitness individuals. The other CBU templates exhibit the opposite, most of the genes yield MOPs with larger inner sphere volumes, leading to the population averages for the (3-pyramidal)_4_(2-linear)_6_ and (3-pyramidal)_4_(3-planar)_4_ starting and remaining above the target volume.

**Figure 4:**
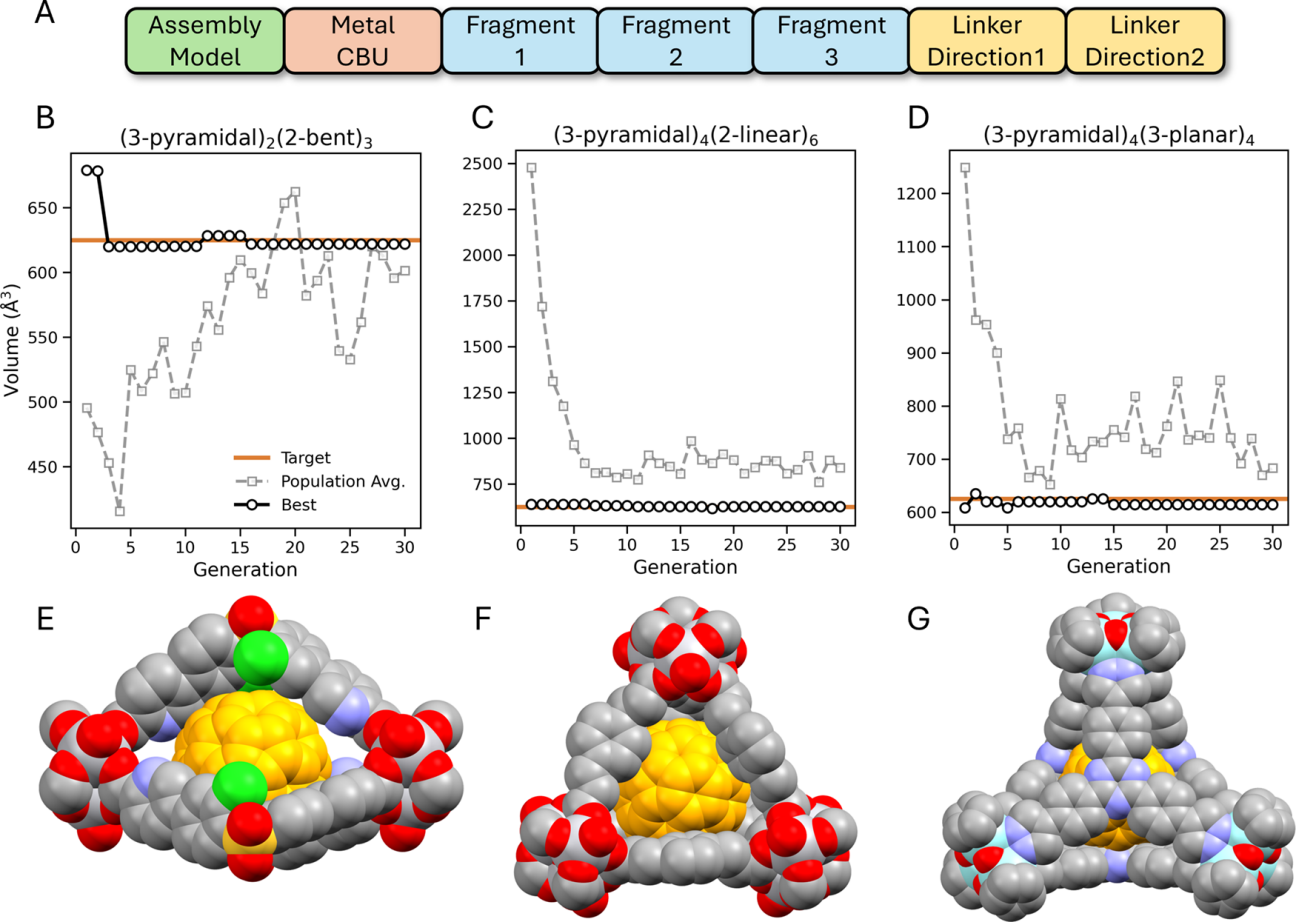
Genetic algorithm optimization of the MOP internal cavity volume to accommodate C_60_. An example gene for a CBU template containing three distinct fragments, two orientated (A), the convergence of the GA trajectories showing the volume of the best ranked and population average at each generation aggregated across the three assembly models (B-D), and the top ranked MOP found for each assembly model encapsulating C_60_ (E-G). The C_60_ atoms are colored orange and hydrogen atoms are omitted for clarity.

Overall, only 3,814 MOP configurations, corresponding to 2.5% of the total search space, were evaluated to identify the optimized structures. This efficiency strongly indicates the effectiveness of GA optimization combined with FragMOPs.

#### 3.2.2 CO_2_ Interaction Energy

As a more complex demonstration of optimization within the FragMOPs space, we next searched for MOPs exhibiting strong CO_2_ interactions, which could be potential candidates for carbon capture applications. We focused on the chemical space surrounding a known CO_2_-binding MOP, MOP-1,^65^ which is assembled from a 2-linear organic CBU containing three symmetrically arranged linker fragments, the [Zr_3_O­(OH)_3_(C_5_H_5_)_3_] metal CBU, and the (3-pyramidal)_2_(2-linear)_3_ assembly model. Extracting all organic CBUs matching this template from the FragMOPs data set yielded 5,912 unique molecules. Combining these organic units with the same metal CBU and assembly model resulted in 936 successfully assembled MOPs, the remainder being removed due to atom overlaps arising from the narrow geometry of the assembly model.

To estimate the CO_2_ interaction energies, we employed a Widom insertion approach, involving Monte Carlo sampling of accessible CO_2_ positions around each MOP and calculating the interaction energy as *U*_int_ = *U*_MOP+CO_2__ – *U*_MOP_ – *U*_CO_2__. This is averaged over all insertions to yield the ensemble average, ⟨*U*_int_⟩. To balance efficiency and accuracy in these simulations, energy calculations were performed with the small UMA model, a universal machine learning interatomic potential (MLIP) trained across multiple domains, including importantly CO_2_ adsorption in MOFs.^66,67^ The high structural and chemical similarity between MOFs and MOPs, provides confidence the model will retain semiquantitative accuracy when applied to our data set. To assess this, we compared isosteric heat of adsorptions (*Q*_st_) calculated using the UMA model against experimentally reported values for two literature MOPs (including MOP-1) with resolved crystal structures, obtaining a mean absolute error of 2.79 kJ mol^–1^ and correct relative ranking (Table S.3). To further probe the model’s accuracy, we sampled a set of CO_2_ insertions and recalculated them using density functional theory (DFT) at the B97–3c level. The UMA model achieves a strong correlation with the resulting interaction energies (*R* ^2^ = 0.996) and an MAE of 0.072 eV (Figure S.17), equivalent to the errors observed for MOFs previously.^67^ While this limited benchmark does not completely establish transferability, it indicates that the UMA model is sufficiently reliable to reproduce adsorption trends within the defined search space, and thus, is suitable for guiding the optimization of CO_2_ interactions. Considering this, we proceeded to calculate ⟨*U*_int_⟩ values for all 936 MOPs.

Because these simulations were performed on isolated MOPs, we further assessed whether the resulting ⟨*U*_int_⟩ values are representative of material performance. Using crystal structure prediction (CSP) methods, we generated solid-state structures with consistent packing for a subset of the MOPs and computed *Q*_st_ values for the corresponding crystals. A regression between the isolated and solid-state results gave a strong correlation (*R* ^2^ = 0.958, Figure S.15), confirming that the isolated ⟨*U*_int_⟩ values serve as a reliable proxy for material performance. Undoubtedly, varying the crystal packing will significantly affect gas adsorption properties. However, it is reasonable to expect that MOPs with the same shape and metal CBU will have similar crystal energy landscapes, this expectation being supported by experimental studies, which often show similar crystal packing for related MOPs.^7^ Therefore, optimizing interaction energy with the isolated MOP will most likely lead to higher material performance.

The distribution of average CO_2_ interaction energies across the 936 assembled MOPs is shown in Figure 5. The calculated ⟨*U*_int_⟩ values range from −0.0828 to −0.326 eV, with a mean of −0.128 eV. MOP-1 exhibits a value of −0.154 eV, and in total 57 MOPs are predicted to have stronger average interactions with CO_2_. While absolute rankings are subject to the uncertainty of the ML potential, analyzing the interaction energies across the data set reveals clear chemical trends that suggest general guiding principles. For example, averaging ⟨*U*_int_⟩ for each fragment across all CBU occurrences shows that more functionalized fragments, especially those involving nitrogen heterocycles, consistently lead to more stable CO_2_ interactions. Interestingly, the top-ranked fragment is the sulfonate group present in MOP-1, though its limited frequency (two MOPs) could be biasing this result. At the other end, fragments with aliphatic or halogen substituents are found to yield the weakest interactions on average. Additionally, comparing the results for the two binding groups reveals that MOPs featuring the pyrazolate fragment have slightly more stable average ⟨*U*_int_⟩ than those featuring the carboxylate fragment, which is found to be statistically significant (Figure S.24). Overall, we find these insights align closely with prior experimental and computational studies that have demonstrated more polarized functionalities tend to increase affinity for CO_2_ due to stabilizing interactions with the CO_2_ quadrupole.^68–74^

**Figure 5:**
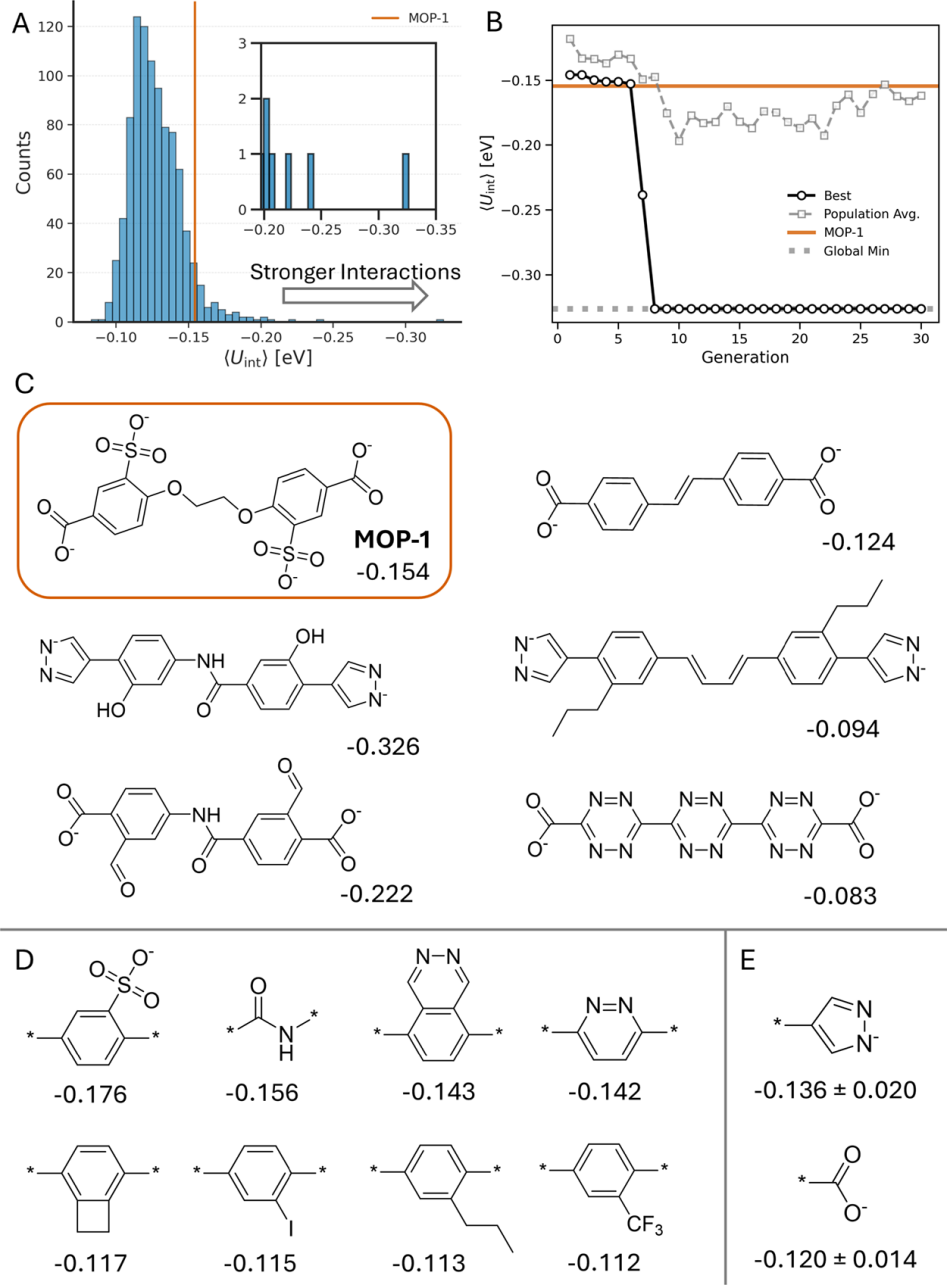
Results from optimizing average CO_2_ interaction energy for (3-pyramidal)_2_(2-linear)_3_ MOPs using organic CBUs containing three linker fragments. The distribution of average CO_2_ interaction energy for all 936 valid MOPs (A) and the trajectory from the GA optimization (B) are shown with reference to the reported CO_2_ MOP host, MOP-1. The CBUs estimated to have the minimum, maximum and median ⟨*U*_int_⟩ (C) and the four fragments with the lowest and highest average ⟨*U*_int_⟩ (D) as well as the average ⟨*U*_int_⟩ and standard deviation for both binding fragments (E) are highlighted.

The results from genetic optimization applied to ⟨*U*_int_⟩ are also summarized in Figure 5. Using a fitness function containing solely ⟨*U*_int_⟩, The GA rapidly converges to the global minimum of the search space within seven generations, and the population mean surpasses MOP-1 after only a further two generations. Considering that many of the chromosomes do not successfully assemble, it is remarkable the algorithm can identify high-performing candidates rapidly in such a fragmented space. Together, these results underscore the synergy in combining the FragMOPs framework with machine-learning–accelerated simulations to efficiently discover and rationalize promising MOPs for gas capture.

## 4 Discussion

The FragMOPs approach presented here has enabled the exploration of a chemical space several orders of magnitude larger than that previously created through direct enumeration of known CBUs and assembly models. This substantial expansion was achieved while maintaining realistic molecules through the selection of simple fragments, templates derived from known CBUs, and enforcing constraints on fragment type and symmetry. Moreover, encoding the molecular fragments as genes was found to be an effective method to optimize the assembled MOPs toward desired properties. These results are a significant advancement yet there is considerable scope to develop and extend the FragMOPs approach further, both in terms of chemical space and methods.

First, this study focused on a select number of assembly models and complementary CBU templates and as such there is clear potential to expand the data set through adding more assembly models and CBU templates. Indeed, the OntoMOPs data set includes 20 assembly models, and therefore, if we extrapolate the results we achieved with the selected four assembly models, it is reasonable to expect that the FragMOPs approach could yield millions of unique MOP configurations. This could be further amplified by adding more fragments, which can also expand the diversity of chemistry and functionalities in the resulting MOPs beyond the relatively simple, singly functionalized linkers that were used here. While generative models have an advantage over fragmentation approaches in that they can explore unconstrained chemical spaces beyond the training data, the results presented clearly illustrate that with sufficient fragments the chemical diversity of the CBUs generated is considerable. Moreover, rather than choosing one or the other, we expect that fragmentation and generative models can be combined synergistically to explore large chemical spaces with a high level of control.

An important consideration when expanding the chemical space is to ensure that the geometries of generated CBUs translate to realistic MOP geometries. In this study, the geometries of fragments were kept rigid and the only flexibility was in the torsions between fragments, which were adjusted to eliminate overlaps between nonbonding atoms. While this guarantees the CBUs will have suitable geometries to function as intended, not adapting the conformation within the MOP environment can lead to collisions between the CBUs, which in the results presented were simply marked as failed assemblies and removed from further analysis. Consequently, with the current approach this can lead to a number of valid MOP configurations not being successfully assembled. For example, in creating the data set of 3000 MOPs in Section 3.1 we recorded 181 failed MOP assemblies. Improving the computational construction of supramolecular assemblies has been demonstrated in previous studies through applying geometry optimization methods, and we expect this would also be effective in the MOP assembler, though careful consideration will be required for how the metal CBUs are modeled as these are not described well by standard force fields such as MMFF94.^36,37,75,76^ Including geometry optimizations would also yield more realistic cavity environments, and thereby, improved downstream evaluation for host–guest applications.

A further refinement that could improve the quality of the results is a stronger focus on the synthesizability, both at the level of the CBU and MOP. While we attempted to incorporate some features of this through assessing the SAscore of CBUs and adding penalties to the fitness function that guided the genetic optimization toward simpler CBUs, these approaches have limitations. For example, SAscore is only an approximate estimate of synthesizability and is less effective for differentiating molecules with similar scores, as is the case with the majority of the FragMOPs CBUs. A potential approach to improve the assessment of CBU synthesizability would be to include retrosynthesis methods to estimate the synthesizability through the number of steps required to reach commercially available reactants, which has been demonstrated to effectively guide reinforcement learning.^77^ By contrast, accounting for MOP synthesizability will require including estimates of the formation energy, which are important not only for indicating if a MOP is thermodynamically favored to form, but also, in cases where multiple assemblies are possible, which will be dominant. These calculations can be complex with results that vary depending on synthesis conditions, however, previous studies have shown the effectiveness of including these aspects in modeling supramolecular assemblies.^78,79^ If possible to efficiently geometry optimize the MOP structures, simpler metrics, such as the strain energy of the organic CBU, could also be used as a proxy for synthesizability.^80^ Furthermore, with our approach the integration of the knowledge graph provides an opportunity to involve semantic reasoning based on experimental observations and heuristics in predicting MOP synthesizability.^7^

## 5 Conclusions

Developing efficient and effective methods to computationally screen materials for advanced applications is a critical area of research toward accelerating materials discovery. In this study we have presented a method for generating an extensive chemical space of organic building units suitable for reticular chemistry based on assembling molecular fragments. We demonstrated applying this method to explore MOPs resulting in the FragMOPs data set containing 98,098 CBUs and encapsulating a configurational space of 791,056 MOPs across four assembly models and seven metal units. Furthermore, we showed that a genetic algorithm applied to the molecular fragments enables effective optimization of the MOP properties, with examples illustrating the optimization of cavity size and CO_2_ interaction energies estimated from machine-learning-accelerated simulations. While the current implementation has limitations in single CBU conformations and synthesizability information, the FragMOPs approach provides a robust foundation for future developments, including expanded fragment libraries, integrated conformational and retrosynthesis analysis, and thermodynamic evaluations. Overall, this work establishes a scalable, explainable, and data-efficient strategy for the accelerated rational design of high-performing reticular materials for applications.

## Supplementary Material



## Data Availability

All codes and ontologies developed are available on GitHub under MIT license: www.github.com/TheWorldAvatar/MOPTools.

## Notes

Generic shapes of the CBUs and MOP assembly models used in this study; diagram of the FragMOPs ontology; illustration of the progression from the definition of a CBU template to an assembled CBU; summary of fragment types and sequence positions for each CBU template defined; summary of the number of unique CBUs assembled for each template; t-SNE plot; distributions of molecular weight; three highest molecular weight CBUs; and other figures (PDF)
