# Supplementary material for: Inverse Design of Metal–Organic Polyhedra through Molecular Fragmentation and Evolutionary Optimisation
Source: J Chem Inf Model. 2026 Apr 1;66(7):3933–43. doi: 10.1021/acs.jcim.5c02956 (PMC13080989; doi:10.1021/acs.jcim.5c02956)
Supplement: Supplementary file 1 [file ci5c02956_si_001.pdf]

Supporting Information
Inverse Design of Metal-Organic Polyhedra through Molecular Fragmentation and Evolutionary Optimisation

Patrick W.V. Butler,[†] Simon D. Rihm,[†] Sebastian Mosbach,[†,‡] Jethro Akroyd,[†,‡] and Markus Kraft[*,†,‡,¶]

†*Department of Chemical Engineering and Biotechnology, University of Cambridge, Philippa Fawcett Drive, Cambridge, CB3 0AS, UK*

‡*CARES, Cambridge Centre for Advanced Research and Education in Singapore, 1 Create Way, CREATE Tower, #05-05, Singapore, 138602*

¶*MIT, Chemical Engineering, 77 Massachusetts Avenue, Room E17-504, Cambridge, MA 02139 USA*

E-mail: mk306@cam.ac.uk

S.1 Computational Methods

Widom Simulations

Estimates of CO_2 Boltzmann-weighted average interaction energy, $\langle U_{\text{int}} \rangle$, and heat of adsorption, Q_{st}, were calculated using the Widom insertion method.[1] These simulations were performed using a modified version of the widom python package,[2] which is based on the DAC-SIM package.[3] The modifications made were to enable a maximum distance from host (i.e. MOP) atoms and allowing for an insertion grid in the same manner as DAC-SIM. All simulations were performed using the UMA-s-1p1 model with the ODAC task setting.[2,4] For average interaction energies, the isolated MOP was first optimised using the ASE[5] FIRE optimizer to a force tolerance of 0.05 eV Å^{-1} and then placed in a box with a vacuum of 5 Å on all sides. The Widom simulation was then conducted with a minimum insertion distance of 1.0 Å between any gas and host atoms and a maximum distance of 3.5 Å from any host atom to the centroid of the gas molecule. Approximately 5000 insertions were calculated for each structure and the average interaction energies were averaged over two independent runs. The procedure was identical for the case of estimating CO_2 heat of adsorption with the exception the number of insertions was increased to 10,000. All simulations were conducted using NVIDIA A100 GPUs with the isolated MOP simulations requiring on average 14.7 minutes of wall-time on one GPU per data point. The modified widom package is available at github.com/pwvbutler/widom.

Crystal Structure Prediction

To generate crystal structures for assembled MOPs we used quasi-random sampling as implemented in mol-CSPy.[6] The code was modified to interface with the GULP implementation of the UFF4MOF force field for geometry optimisations.[7–9] After generating trial crystal structures from the MOP geometry and the appropriate number of chloride counter ions, the cell was optimised with an applied pressure of 0.1 GPa and the rigid-body constraint.

The structures were then subsequently optimised without the rigid-body constraint to a maximum force tolerance of 0.01 eV Å^{-1} using the ASE FIRE optimizer and the UMA-s-1p1 ASE calculator with the ODAC task setting.[2,4] The modified mol-CSPy code is available at gitlab.com/pwvbutler/mol-cspy.

DFT Calculations

Calculations of select interaction energies between CO_2 and MOP hosts were performed using the B97-3c method[10] implemented in ORCA 4.2.1.[11] These involved single point calculations of the isolated MOP, CO_2 molecule, and MOP-CO_2 complex, with the MOP structures optimised by the UMA-s-1p1 workflow described above. A Self-Consistent Field (SCF) convergence threshold of 1.0×10^{-6} Eh was used for all calculations.

S.2 CBU and Assembly Model Shapes

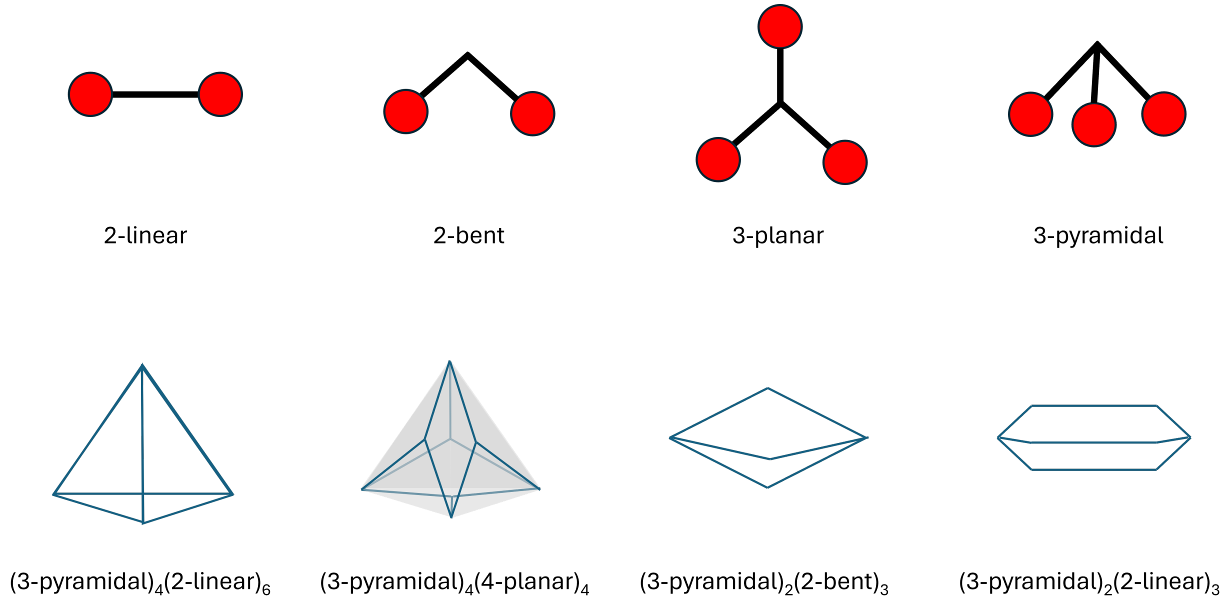

Figure S.1: The generic shapes of the CBUs and MOP assembly models used in this study.

S.3 Molecular Fragment SMILES

Binding Groups

O=[C]([O-])[*]
[*][c]1cn[n-]c1

Linkers

[*]/[CH]=[CH]/[*]
[*][CH2][*]
[*][c]1cc[c]([*])cc1
O=[S](=O)([*])[*]
[*][C]#[C][*]
[*]/[N]=[N]/[*]
[*][c]1cc[c]([*])c2ccccc12
[*][c]1cc[c]([*])nc1
[*][c]1cc2c3c(c1)CCc1c[c]([*])cc(c1-3)CC2
[*][c]1c2ccccc2[c]([*])c2ccccc12
[*][O]CC[O][*]
[*]/[CH]=C/C=[CH]/[*]
[*][c]1nn[c]([*])nn1
[*][c]1cc2ccc3c4ccc5c[c]([*])cc6ccc(c7ccc(c1)c2c37)c4c56
[*][c]1cc2cc3ccc4cc5c[c]([*])cc6cc7ccc8cc(c1)c2c1c3c4c(c56)c7c81
[*][c]1ccc2c(ccc3c[c]([*])ccc32)c1

```
[*][c]1ccc2c(cnc3c[c]([*])ccc32)c1

[*][c]1c2nc(cc3ccc([nH]3)[c]([*])c3nc(cc4ccc1[nH]4)C=C3)C=C2

[*][c]1cc2ccc3c[c]([*])cc4ccc(c1)c2c34

[*][CH2][CH2][*]

[*][CH2][O][*]

O=[C]([*])[NH][*]

[*][C]#CC#[C][*]

[*][C]#CC#CC#[C][*]

[*][C]#C/C=C/C#[C][*]

[*]/[CH]=[N]/[*]

[*][C@]12CC[C@]([*])(CC1)CC2

[*][C@]12[C@@H]3[C@@H]4[C@H]1[C@@H]1[C@H]2[C@H]3[C@]41[*]

[*][c]1cc[c]([*])c2cnncc12

[*][c]1cc[c]([*])c2c1OCO2

[*][C@]12c3ccccc3[C@]([*])(c3ccccc31)c1ccccc12

CSC/[C]([*])=[C](\[*])CSC

O=[C]([*])[*]

[*][O]CC[O][*]

[*][c]1cc[c]([*])c2c1CC2

[*][c]1cnc2c(ccc3c[c]([*])cnc32)c1

[*][O][*]

F[C](F)([*])[*]
```

O=C1C=[C]([*])C(=O)C=[C]1[*]
[*][c]1cn[c]([*])cn1
[*][c]1cc2s[c]([*])cc2s1
[*][C@H]1C[C@@H]2[C@H]3C[C@H]([*])C[C@H]3[C@@H]2C1
[*][c]1cc[c]([*])nn1

Nodes

[*][c]1c[c]([*])c[c]([*])c1
[*][c]1n[c]([*])n[c]([*])n1

Side Chains

[F][*]
OCC[CH2][*]
OCC[O][*]
O=[S](=O)([O-])[*]
CC[CH2][*]
[Br][*]
C[CH](C)[*]
[NH2][*]
[Cl][*]
[*][c]1ccccc1

C[C](C)(C)[*]
C[O][*]
CC[O][*]
O=[N+]([O-])[*]
[OH][*]
OCC[O][*]
[CH3][*]
[I][*]
C[CH2][*]
CO[CH2][*]
N#[C][*]
[SH][*]
F[C](F)(F)[*]
O=[CH][*]

S.4 FragMOPs Ontology

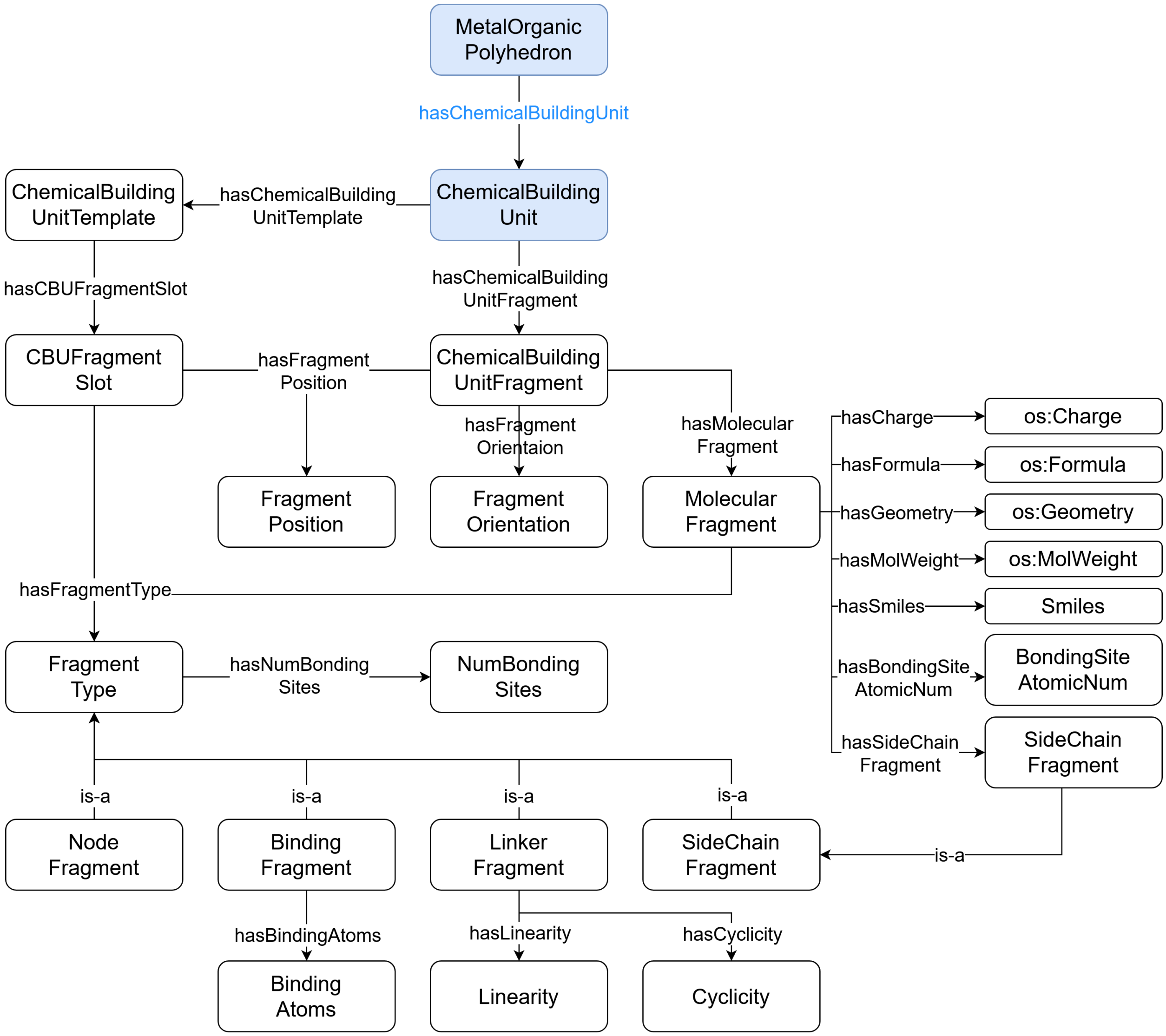

Figure S.2: Diagram of the FragMOPs extensions from the base OntoMOPs MetalOrganicPolyhedron and ChemicalBuildingUnit concepts (coloured blue) and OntoSpecies (abbreviated os).

S.5 CBU Assembler

S.5.1 Overview

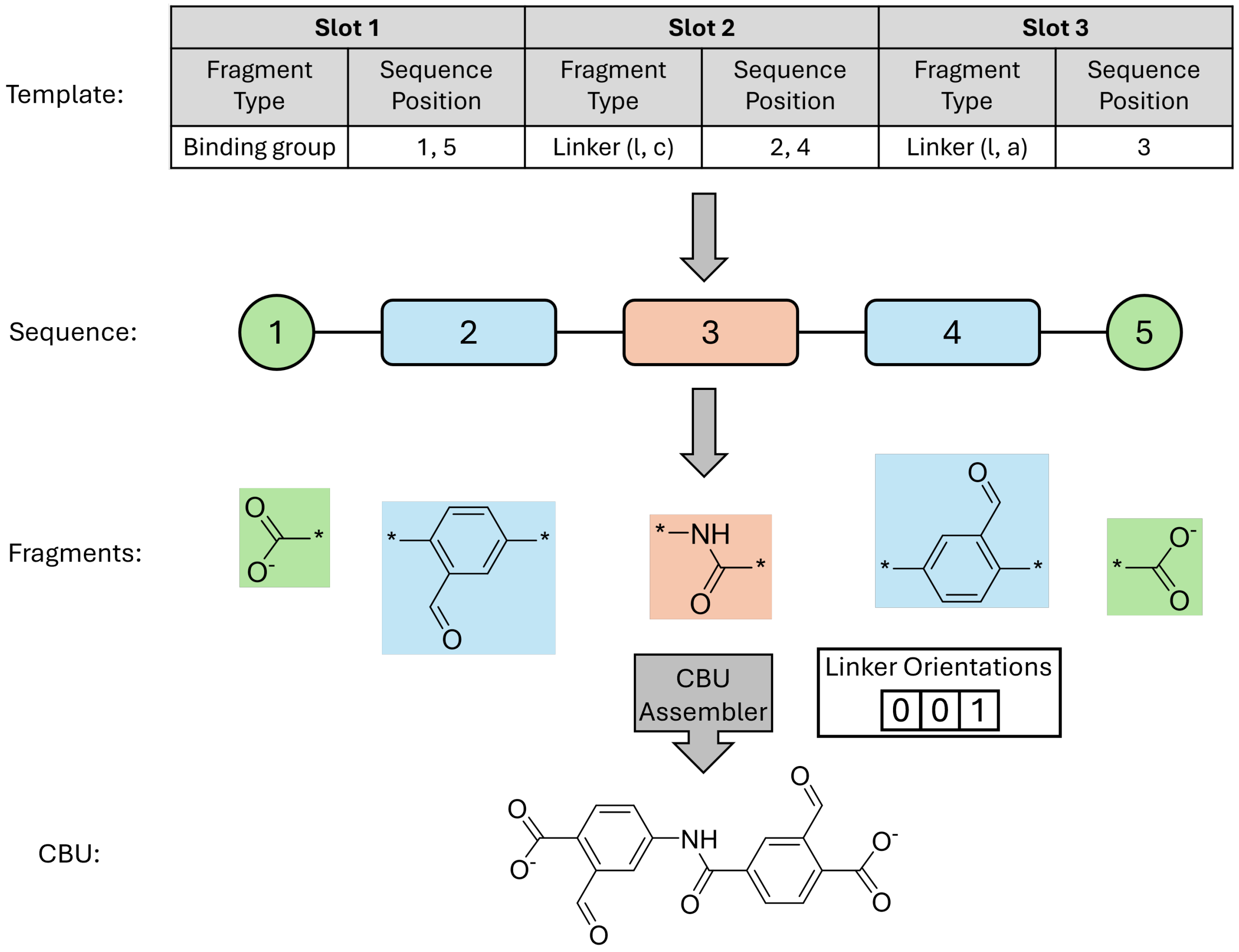

Figure S.3: Illustration of the progression from the definition of a CBU template to an assembled CBU. The colours correspond to the template slots and thereby the specific fragment types required for the slot. The position values specify the sequence for joining fragments and is only strictly required for linker slots, binding group and node fragments always being joined at the ends of the sequence. The orientations of linkers are set by a vector with values of 0 or 1 corresponding to the linker sequence. Legend: l = linear, c = cyclic, a = acyclic.

S.5.2 CBU Templates

Table S.1: Summary of fragment types and sequence positions for each CBU template defined. With the exception of the 2-linear template 1, cyclic linkers with more than 12 heavy atoms are excluded from the accepted fragments. The position values specify the sequence for joining fragments and is only strictly required for linker slots, binding group and node fragments always being joined at the ends of the sequence. Legend: c = cyclic, a = acyclic, l = linear, n = non-linear.

**Template**	**Slot 1**		**Slot 2**		**Slot 3**		**Slot 4**	
	**Fragment type**	**Pos.**	**Fragment type**	**Pos.**	**Fragment type**	**Pos.**	**Fragment type**	**Pos.**
2-linear template 1	binding group	1, 3	linker (l, c/a)	2	–	–	–	–
2-linear template 2	binding group	1, 4	linker (l, c)	2	linear linker (l, c)	3	–	–
2-linear template 3	binding group	1, 5	linker (l, c)	2, 4	linker (l, c/a)	3	–	–
2-linear template 4	binding group	1, 4	linker (l, a)	2, 4	linker (l, c)	3	–	–
2-bent template 1	binding group	1, 5	linker (l, c)	2, 4	linker (n, a)	3	–	–
2-bent template 2	binding group	1, 7	linker (l, c)	2, 6	linker (l, c)	3, 5	linker (n, a)	4
3-planar template 1	binding group	3	linker (l, c)	2	node (3)	1	–	–
3-planar template 2	binding group	4	linker (l, c)	3	linker (l, c)	2	node (3)	1

Table S.2: Summary of the number of unique CBUs assembled for each template.

**Template**	**Number of CBUs**	
	**Unconstrained**	**Symmetric**
2-linear template 1	120	120
2-linear template 2	3422	116
2-linear template 3	9660	5916
2-linear template 4	1708	1360
2-bent template 1	820	580
2-bent template 2	73000	33640
3-planar template 1	136	136
3-planar template 2	9232	9232
**Total**	98098	51100

S.5.3 Commercially Available FragMOPs CBUs

```
O=C(O)c1nnc(C(=O)O)nn1

O=C(O)CCC(=O)O

O=C(O)c1ccc(C(=O)O)nn1

O=C(O)c1cnc(C(=O)O)cn1

O=C(O)C#CC(=O)O

Nc1cc(C(=O)O)ccc1C(=O)O

O=C(O)c1ccc(C(=O)O)c(O)c1

O=Cc1cc(C(=O)O)ccc1C(=O)O

O=C(O)c1ccc(C(=O)O)nc1

N#Cc1cc(C(=O)O)ccc1C(=O)O

O=C(O)c1ccc(C(=O)O)c(F)c1

O=C(O)c1ccc(C(=O)O)cc1

COc1cc(C(=O)O)ccc1C(=O)O

Cc1cc(C(=O)O)ccc1C(=O)O

O=C(O)CCc1cnc(CCC(=O)O)cn1

O=C(O)COc1ccc(OCC(=O)O)cc1

O=C(O)c1ccc(C(=O)O)c([N+](=O)[O-])c1

O=C(O)CCc1ccc(CCC(=O)O)cc1

O=C(O)c1ccc(-c2ccc(C(=O)O)nc2)cn1

O=C(O)c1ccc(C(=O)O)c(Cl)c1

O=C(O)c1ccc(-c2ccc(C(=O)O)cn2)nc1
```

```
O=C(O)c1ccc(C(=O)O)c(Br)c1

O=C(O)C=Cc1ccc(C=CC(=O)O)cc1

c1cc(-c2cn[nH]c2)ncc1-c1cn[nH]c1

c1cc(-c2cn[nH]c2)ccc1-c1cn[nH]c1

O=C(O)c1ccc(C(=O)O)c(C(F)(F)F)c1

O=C(O)c1cc2sc(C(=O)O)cc2s1

O=C(O)c1ccc(C(=O)O)c2ccccc12

O=C(O)c1ccc(-c2ccc(C(=O)O)cn2)cc1

O=C(O)c1ccc(-c2ccc(C(=O)O)nc2)cc1

CC(C)(C)c1cc(C(=O)O)ccc1C(=O)O

O=C(O)c1ccc(C(=O)O)c(-c2ccccc2)c1

O=C(O)c1ccc(-c2ccc(C(=O)O)cc2)cc1

N#Cc1cc(NC(=O)C(=O)O)ccc1NC(=O)C(=O)O

O=C(O)COc1ccc(OCC(=O)O)c([N+](=O)[O-])c1

Nc1cc(C(=O)O)ccc1-c1ccc(C(=O)O)cc1N

O=C(O)c1ccc(C(=O)O)c(I)c1

O=C(O)COc1ccc(OCC(=O)O)c(Cl)c1

Nc1cc(-c2ccc(C(=O)O)c(N)c2)ccc1C(=O)O

O=C(O)c1ccc(-c2ccc(C(=O)O)c(O)c2)cc1

O=C(O)c1ccc(C(=O)c2ccc(C(=O)O)cc2)cc1

O=C(O)c1ccc(-c2ccc(C(=O)O)cc2O)c(O)c1

Nc1cc(-c2ccc(C(=O)O)cc2)ccc1C(=O)O
```

Nc1cc(C(=O)O)ccc1-c1ccc(C(=O)O)cc1

O=C(O)c1ccc(NC(=O)c2ccc(C(=O)O)cc2)cc1

O=C(O)c1ccc(-c2ccc(C(=O)O)cc2O)cc1

O=C(O)c1ccc(-c2ccc(C(=O)O)c(O)c2)cc1O

O=C(O)c1ccc(C#CC#Cc2ccc(C(=O)O)cc2)cc1

O=C(O)c1cnc2c(ccc3cc(C(=O)O)cnc32)c1

O=C(O)c1ccc(C#Cc2ccc(C(=O)O)cc2)cc1

N#Cc1cc(C(=O)O)ccc1-c1ccc(C(=O)O)cc1C#N

O=C(O)c1ccc(Oc2ccc(C(=O)O)cc2)cc1

O=C(O)c1ccc(-c2ccc(C(=O)O)cc2F)cc1

O=C(O)c1ccc(Cc2ccc(C(=O)O)cc2)cc1

O=C(O)c1ccc(COc2ccc(C(=O)O)cc2)cc1

O=C(O)c1ccc(-c2ccc(C(=O)O)cc2[N+](=O)[O-])cc1

O=C(O)c1ccc(CCc2ccc(C(=O)O)cc2)cc1

O=C(O)CCc1ccc(CCC(=O)O)c2ccccc12

O=C(O)c1ccc(-c2ccc(C(=O)O)c(F)c2)cc1

O=C(O)c1ccc(C=Nc2ccc(C(=O)O)cc2)cc1

Cc1cc(C(=O)O)ccc1-c1ccc(C(=O)O)cc1C

Cc1cc(C(=O)O)ccc1-c1ccc(C(=O)O)c(F)c1

Cc1cc(C(=O)O)ccc1-c1ccc(C(=O)O)cc1

Cc1cc(-c2ccc(C(=O)O)c(C)c2)ccc1C(=O)O

O=C(O)c1ccc(-c2ccc(C(=O)O)c(F)c2)cc1F

```
O=C(O)c1ccc(-c2ccc(C(=O)O)c(Cl)c2)cc1

O=C(O)c1ccc(C=Cc2ccc(C(=O)O)cc2)cc1

O=C(O)c1ccc(N=Nc2ccc(C(=O)O)cc2)cc1

O=C(O)c1c2ccccc2c(C(=O)O)c2ccccc12

O=C(O)c1cc2ccc3cc(C(=O)O)cc4ccc(c1)c2c34

O=C(O)c1ccc(-c2ccc(C(=O)O)c(Cl)c2)cc1F

O=C(O)c1ccc(S(=O)(=O)c2ccc(C(=O)O)cc2)cc1

O=C(O)COc1ccc(OCC(=O)O)c(Br)c1

O=C(O)c1ccc(-c2nnc(-c3ccc(C(=O)O)cc3)nn2)cc1

O=C(O)c1ccc(OCCOc2ccc(C(=O)O)cc2)cc1

O=C(O)c1ccc(-c2ccc(C(=O)O)c(S)c2)cc1S

O=C(O)c1ccc(-c2ccc(-c3ccc(C(=O)O)nc3)cc2)cn1

O=C(O)c1ccc(-c2ccc(-c3ccc(C(=O)O)cc3)nc2)cc1

O=C(O)c1ccc(-c2ccc(-c3ccc(C(=O)O)cc3)cc2)cc1

c1ccc2c(-c3cn[nH]c3)c3ccccc3c(-c3cn[nH]c3)c2c1

Nc1cc(-c2ccc(C(=O)O)cc2)ccc1-c1ccc(C(=O)O)cc1

Nc1cc(-c2ccc(-c3ccc(C(=O)O)c(N)c3)cc2)ccc1C(=O)O

CCOc1cc(-c2ccc(C(=O)O)c(OCC)c2)ccc1C(=O)O

Cc1cc(-c2ccc(C(=O)O)cc2)ccc1-c1ccc(C(=O)O)cc1

O=C(O)c1ccc(-c2ccc(C(=O)O)c3ccccc23)c2ccccc12

Cc1cc(C(=O)O)ccc1-c1ccc(-c2ccc(C(=O)O)cc2C)cc1

COc1cc(C(=O)O)ccc1OCCOc1ccc(C(=O)O)cc1OC
```

```
Nc1cc(-c2ccc(C(=O)O)c(O)c2)ccc1-c1ccc(C(=O)O)c(O)c1

O=C(O)c1ccc(-c2ccc(-c3ccc(C(=O)O)c(O)c3)cc2)cc1O

O=C(O)c1ccc(S(=O)(=O)c2ccc(C(=O)O)c([N+](=O)[O-])c2)cc1[N+](=O)[O-]

O=C(O)c1ccc(-c2ccc(C(=O)O)cc2C(F)(F)F)c(C(F)(F)F)c1

O=C(O)c1ccc(-c2ccc(C(=O)O)c(C(F)(F)F)c2)cc1C(F)(F)F

O=C(O)c1ccc(-c2ccc(-c3ccc(C(=O)O)c(O)c3)c3ccccc23)cc1O

O=C(O)c1ccc(-c2nc(-c3ccc(C(=O)O)cc3)nc(-c3ccc(C(=O)O)cc3)n2)cc1

O=C(O)c1ccc(-c2cc(-c3ccc(C(=O)O)cc3)cc(-c3ccc(C(=O)O)cc3)c2)cc1

O=C(O)c1ccc(-c2ccc(C(=O)O)cc2I)c(I)c1

O=C(O)c1ccc(-c2ccc(-c3nc(-c4ccc(-c5ccc(C(=O)O)cc5)cc4)nc(-c4ccc
        (-c5ccc(C(=O)O)cc5)cc4)n3)cc2)cc1

O=C(O)c1ccc(-c2ccc(-c3cc(-c4ccc(-c5ccc(C(=O)O)cc5)cc4)cc(-c4ccc
        (-c5ccc(C(=O)O)cc5)cc4)c3)cc2)cc1

O=C(O)c1ccc(-c2cc(-c3ccc(C(=O)O)c4ccccc34)cc(-c3ccc(C(=O)O)c4cc
        ccc34)c2)c2ccccc12

c1cc(-c2cc(-c3ccc(-c4cn[nH]c4)cc3)cc(-c3ccc(-c4cn[nH]c4)cc3)c2)
        ccc1-c1cn[nH]c1
```

S.6 CBU Property Distributions

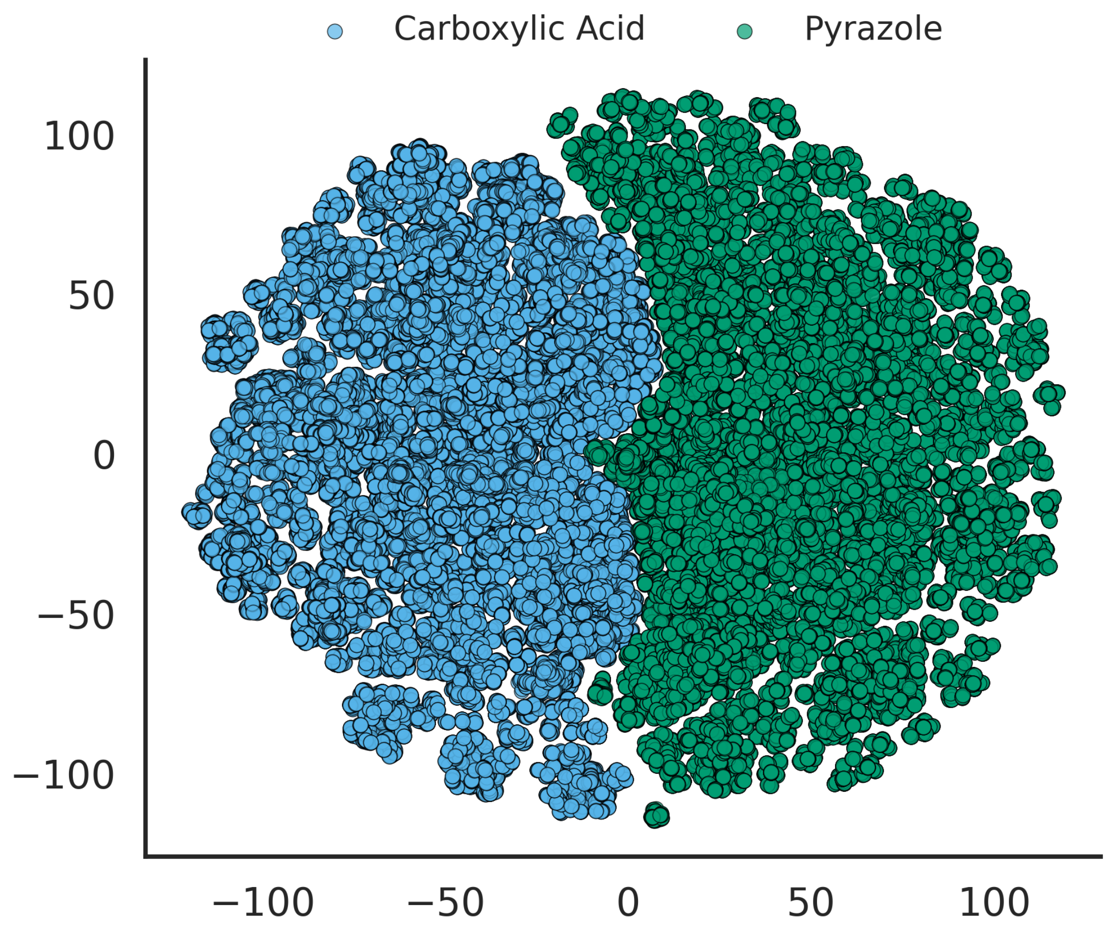

Figure S.4: t-SNE plot of the 2048-bit Morgan fingerprints calculated for the generated FragMOPs CBUs coloured by the binding group being either carboxylic acid or pyrazole.

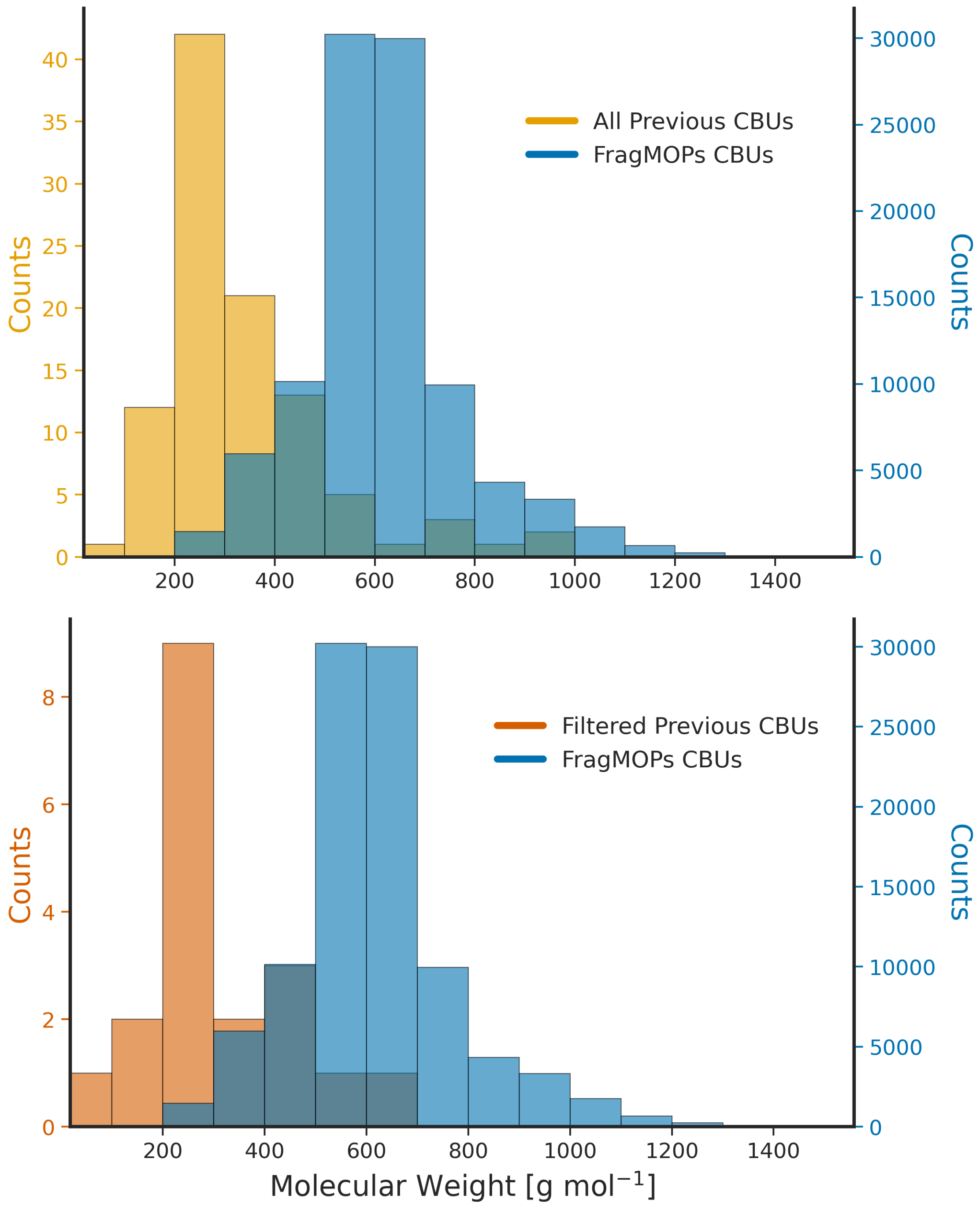

Figure S.5: Distributions of molecular weight across all 101 CBUs from the previous OntoMOPs dataset (above) and the 19 CBUs in the FragMOPs chemical space (below) compared with the FragMOPs CBUs.

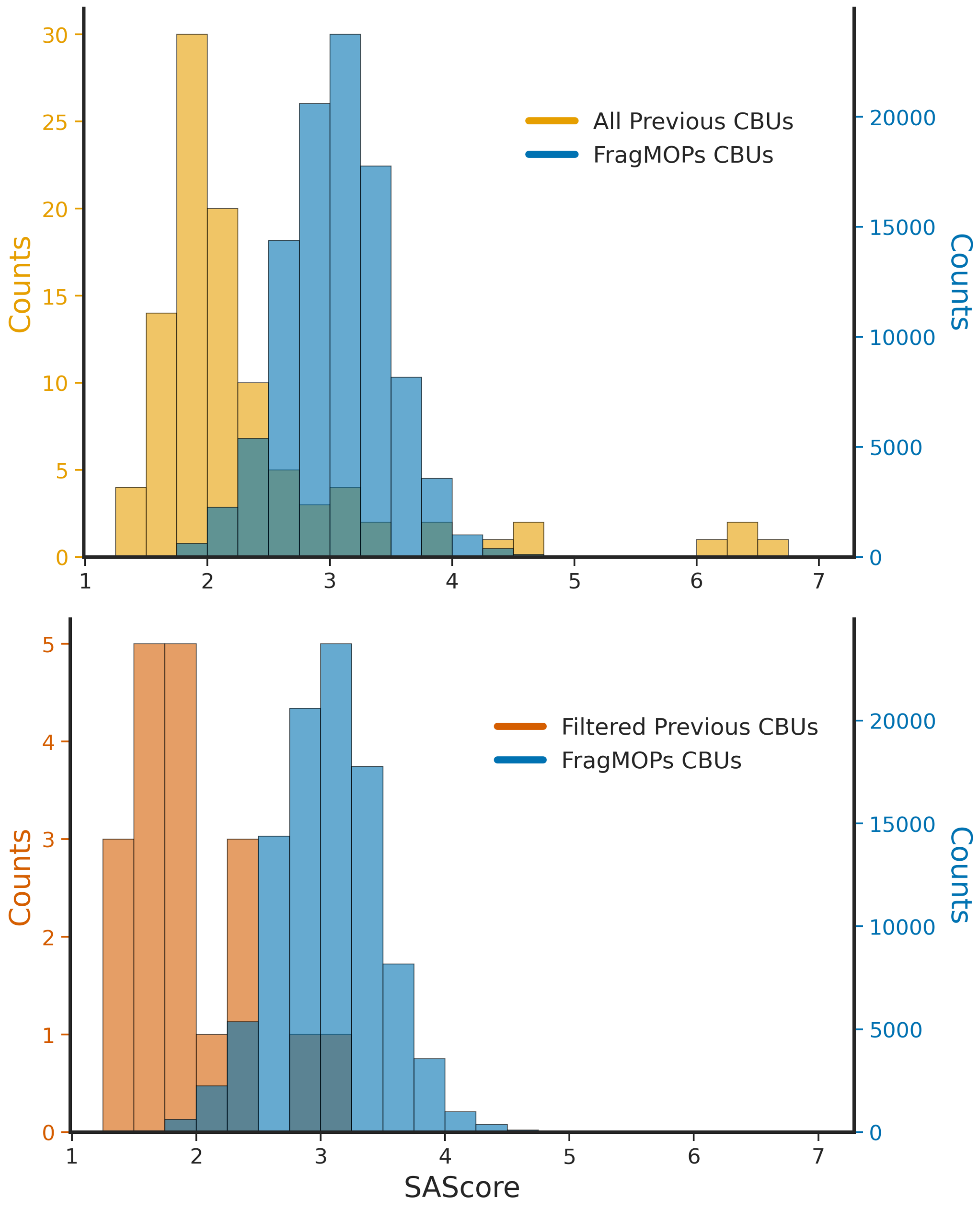

Figure S.6: Distributions of SAscore across all 101 CBUs from the previous OntoMOPs dataset (above) and the 19 CBUs in the FragMOPs chemical space (below) compared with the FragMOPs CBUs. The SAscore values were calculated for the neutral forms of the CBUs.

S.7 Example CBUs

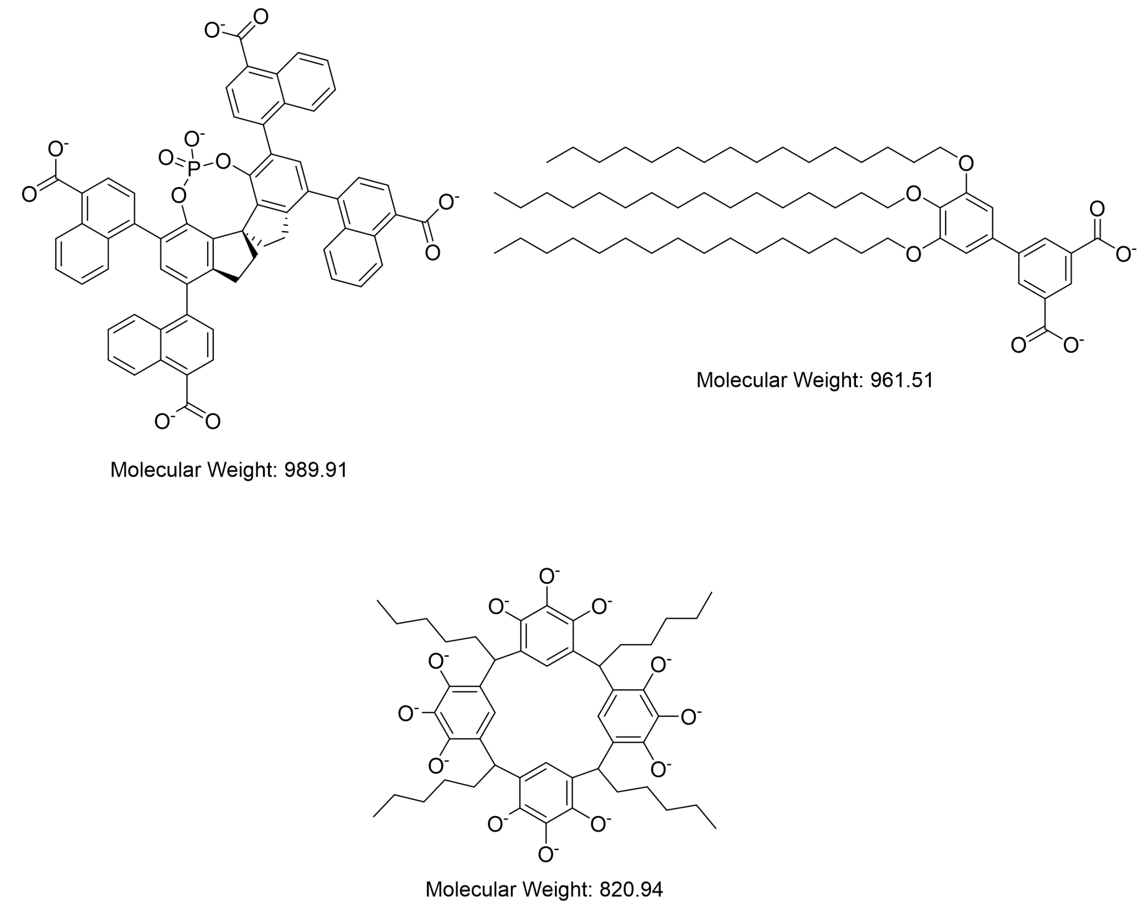

Figure S.7: The three highest molecular weight CBUs in the OntoMOPs dataset.

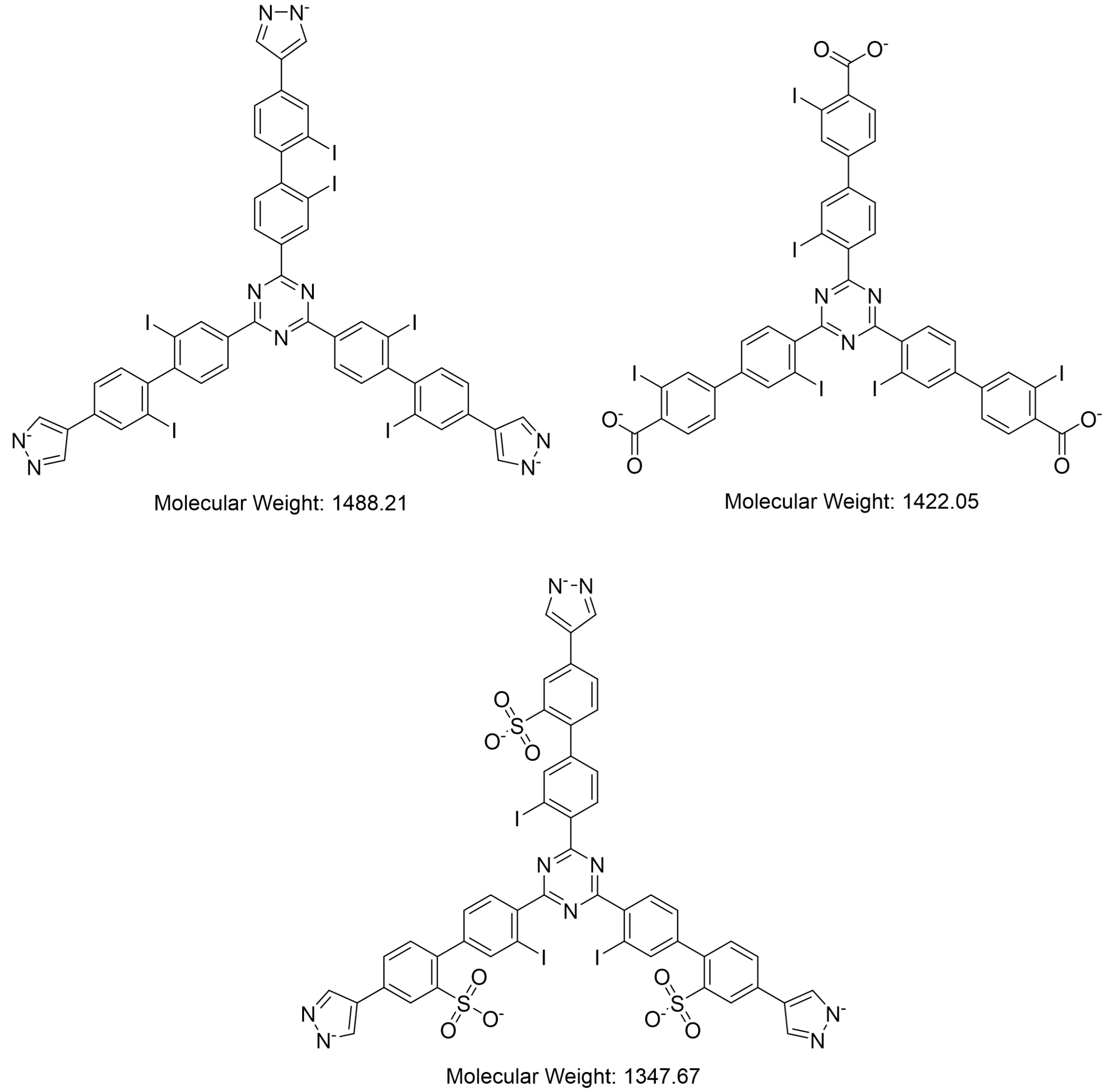

Figure S.8: The three highest molecular weight CBUs in the FragMOPs dataset (excluding isomers).

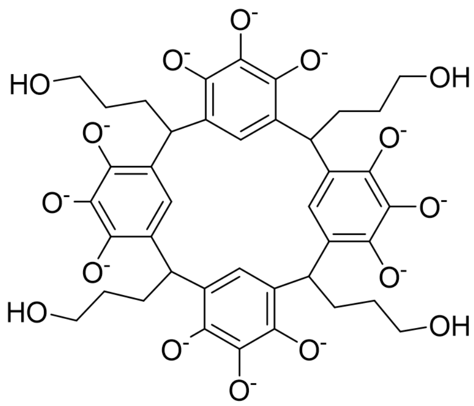

SAscore: 6.50

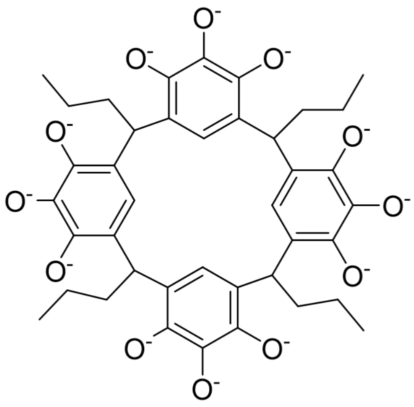

SAscore: 6.45

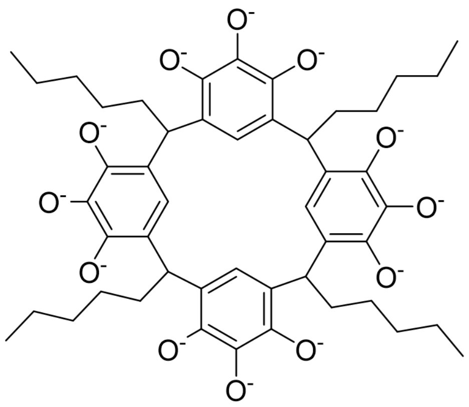

SAscore: 6.31

Figure S.9: The three highest SAscore CBUs in the OntoMOPs dataset. The SAscore was calculated on the charge neutral form.

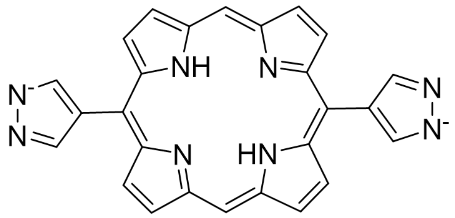

SAscore: 5.85

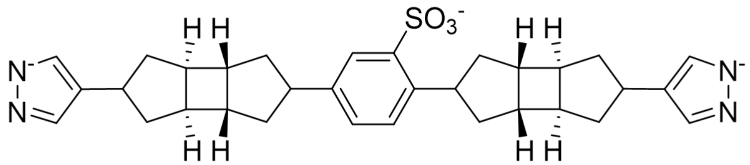

SAscore: 5.66

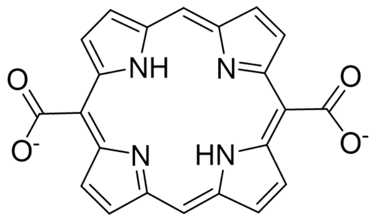

SAscore: 5.65

Figure S.10: The three highest SAscore CBUs in the FragMOPs dataset. The SAscore was calculated on the charge neutral form.

S.8 MOP Cavity Optimisation

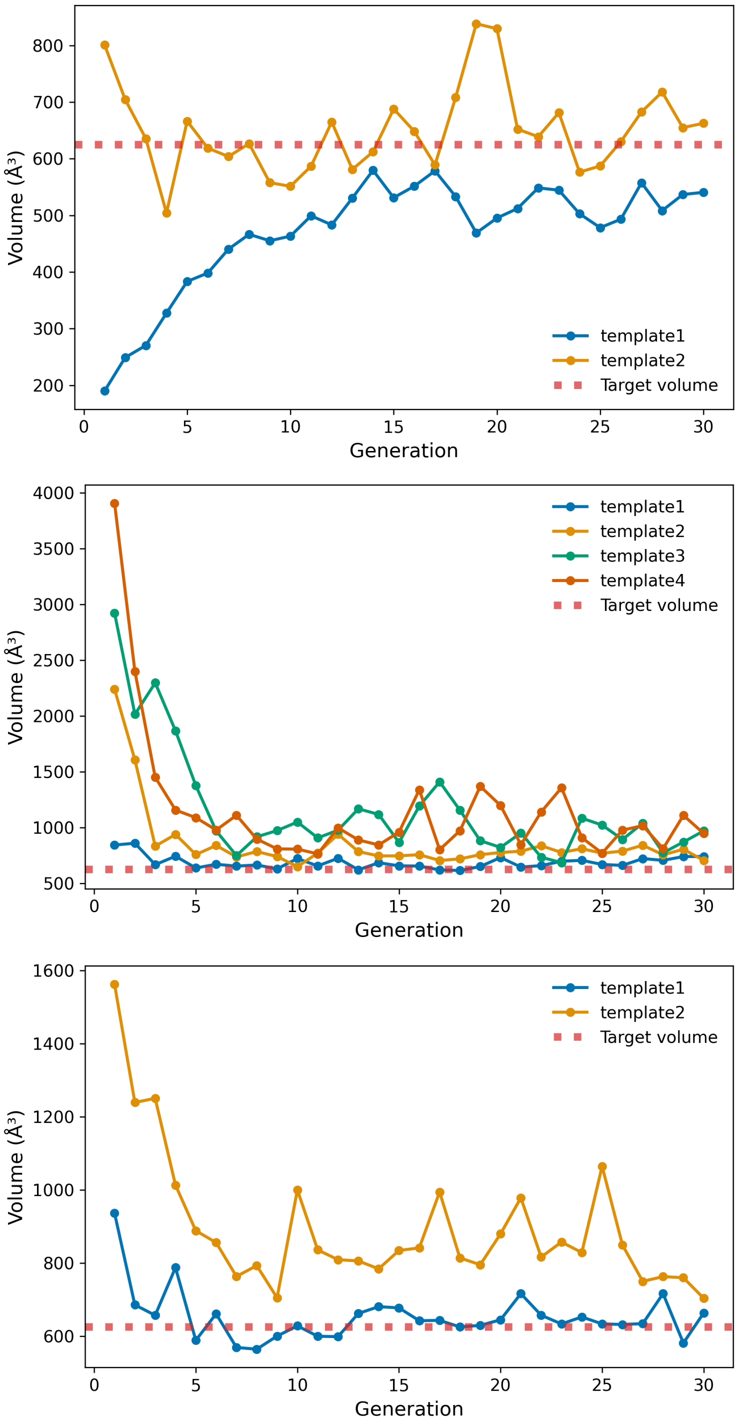

Figure S.11: Population average volumes per CBU template over the genetic algorithm optimisation of the MOP cavity volume to the target volume. Separated by assembly model: (3-pyramidal)$_2$(2-bent)$_3$ (top), (3-pyramidal)$_4$(2-linear)$_6$ (middle), and (3-pyramidal)$_4$(3-planar)$_4$ (bottom).

S.9 Genetic Algorithm Sampled MOPs

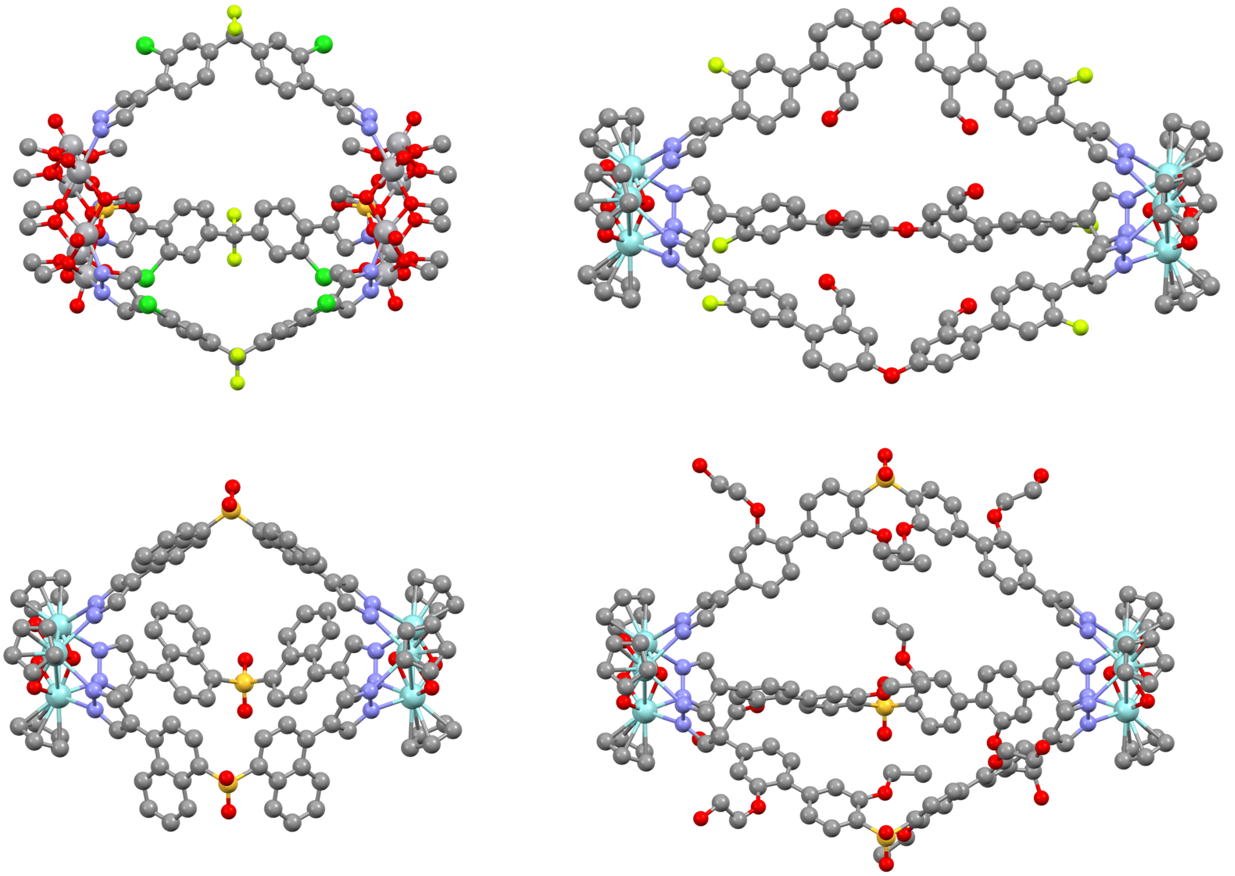

Figure S.12: Select sampled (3-pyramidal)$_2$(2-bent)$_3$ assembled during the genetic algorithm optimisation for C60 encapsulation.

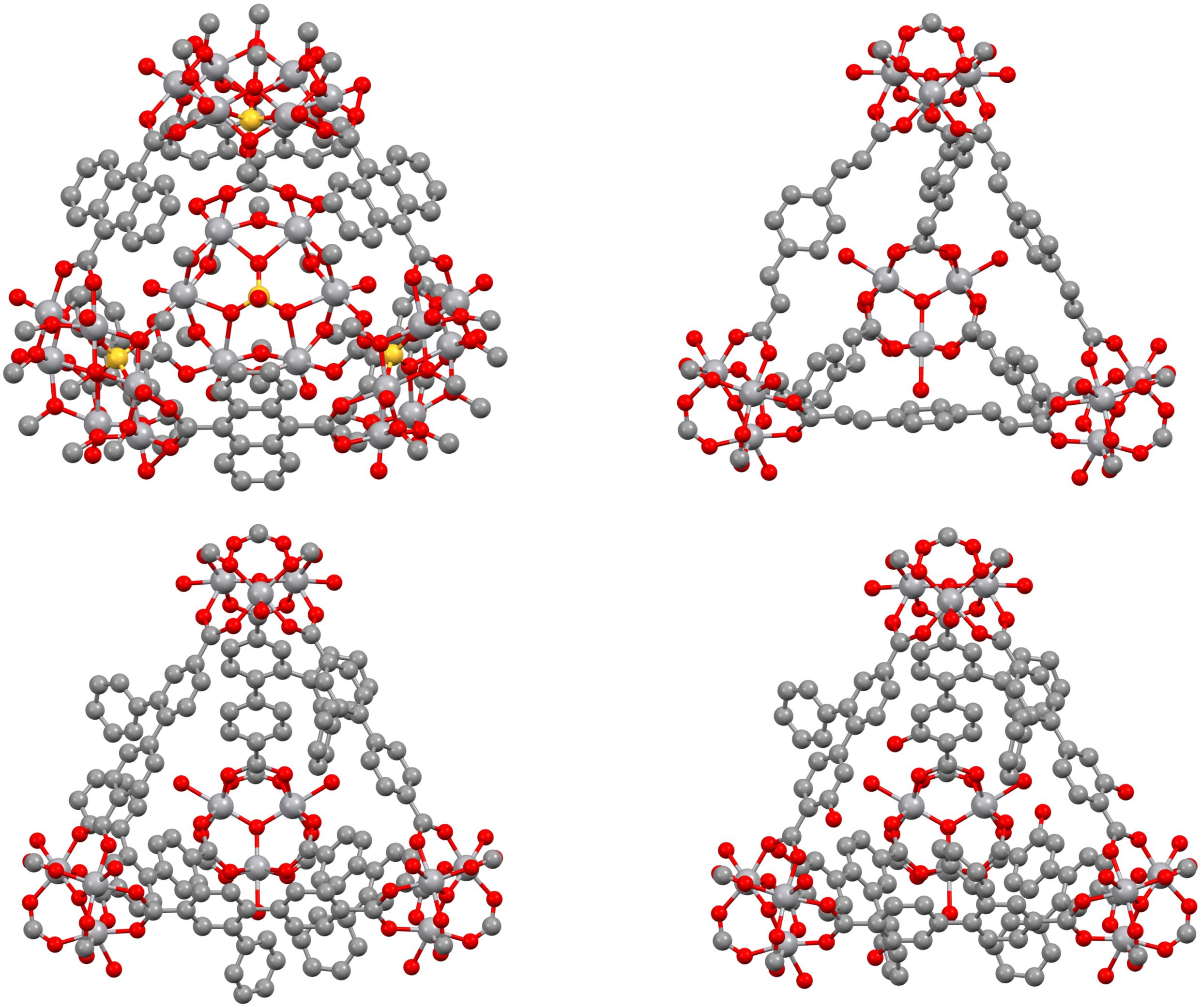

Figure S.13: Select sampled (3-pyramidal)$_4$(2-linear)$_6$ assembled during the genetic algorithm optimisation for C60 encapsulation.

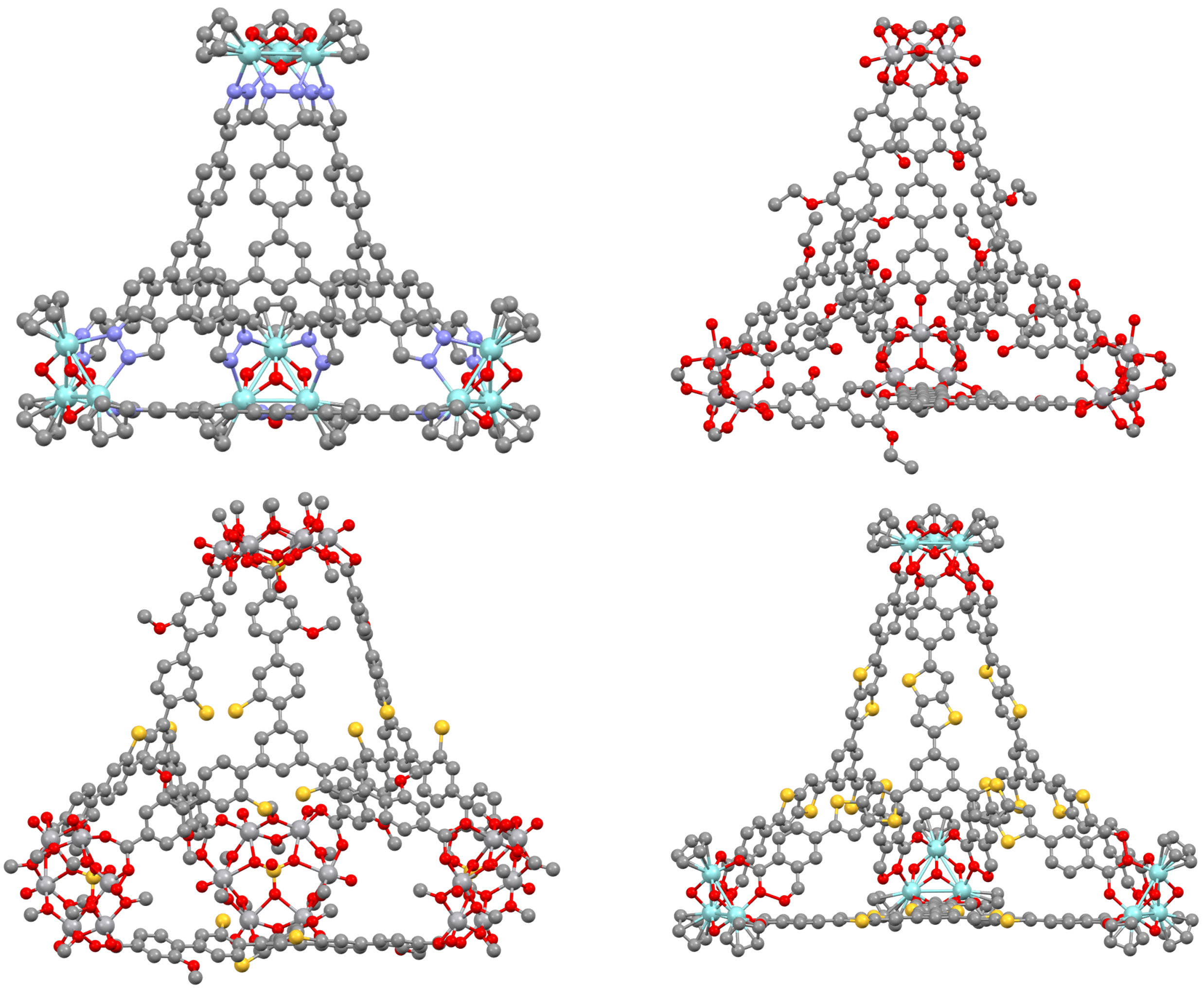

Figure S.14: Select sampled (3-pyramidal)$_4$(3-planar)$_4$ assembled during the genetic algorithm optimisation for C60 encapsulation.

S.10 Simulated Solid-State CO_2 Adsorption

S.10.1 Heat of Adsorption Benchmark

Table S.3: Reported and simulated CO_2 heats of adsorption for experimentally characterised MOPs. The simulated values were determined from Widom simulations using the UMA-s-1p1 ML potential with the ODAC task setting and 10,000 insertions averaged over two independent repeats. Energies in $kJ\,mol^{-1}$. KEXZAR is referred to as MOP-1 in the main text.

CSD Refcode	MOP Formula	Reported	Simulated
KEXZAR	$[Zr_3O(OH)_3(C_5H_5)_3]_2$ $[C_2H_4O_2(C_6H_3SO_3)_2(CO_2)_2]_3$	-24	-28.72
VIBZAK	$[Zr_3O(OH)_3(C_5H_5)_3]_2$ $[SO_2(C_6H_4)_2(CO_2)_2]_3$	-30	-29.13

S.10.2 Correlation between Isolated Average Interaction Energy and Crystal Heat of Adsorption

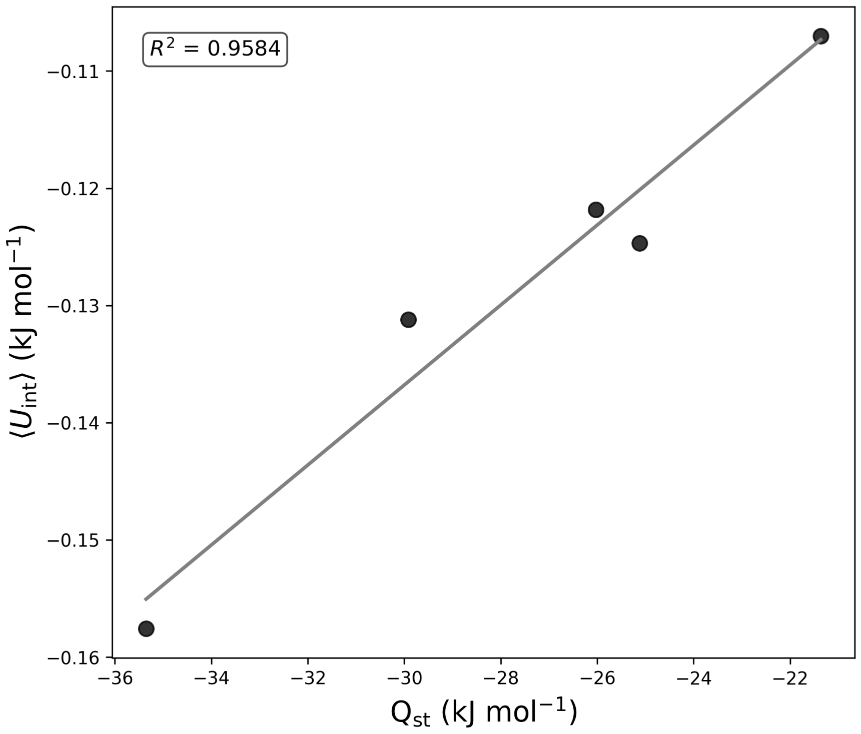

Figure S.15: Correlation between the average CO_2 interaction energy calculated from 5 isolated MOP structures and the heat of adsorption calculated from crystal structures generated by CSP sampling of the isolated MOPs in the $P\bar{1}$ space group using a fixed seed to maintain consistent crystal packing.

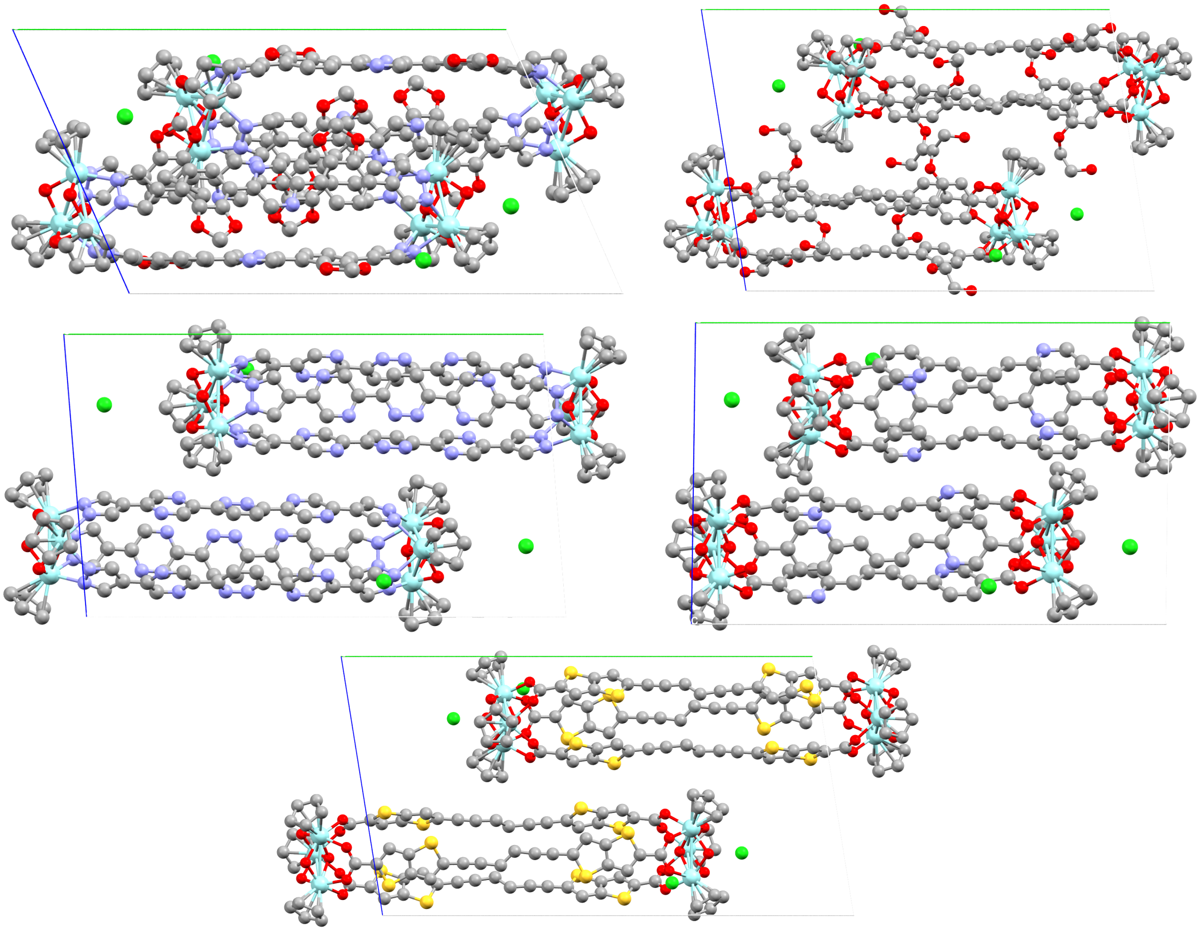

Figure S.16: Unit cells of the FragMOPs generated by crystal structure prediction in the P$\bar{1}$ space group using a fixed seed.

S.11 DFT vs. UMA-s-1p1 CO_2 Interaction Energies

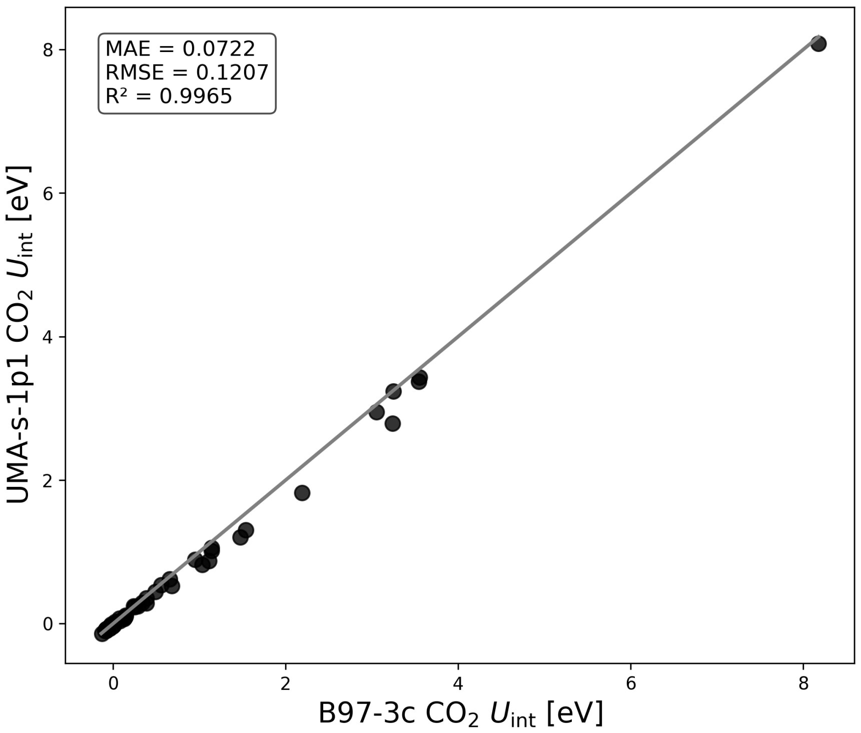

Figure S.17: Comparison of CO_2 interaction energies, U_{int}, estimated by DFT at the B97-3c level and the UMA-s-1p1 model. A total of 49 insertions were sampled across 10 MOPs with 2-linear organic CBUs containing three symmetrically arranged linker fragments, the $[Zr_3O(OH)_3(C_5H_5)_3]$ metal CBU, and the $(3\text{-pyramidal})_2(2\text{-linear})_3$ assembly model.

S.12 Molecular Fragment Average CO_2 Interaction Energy

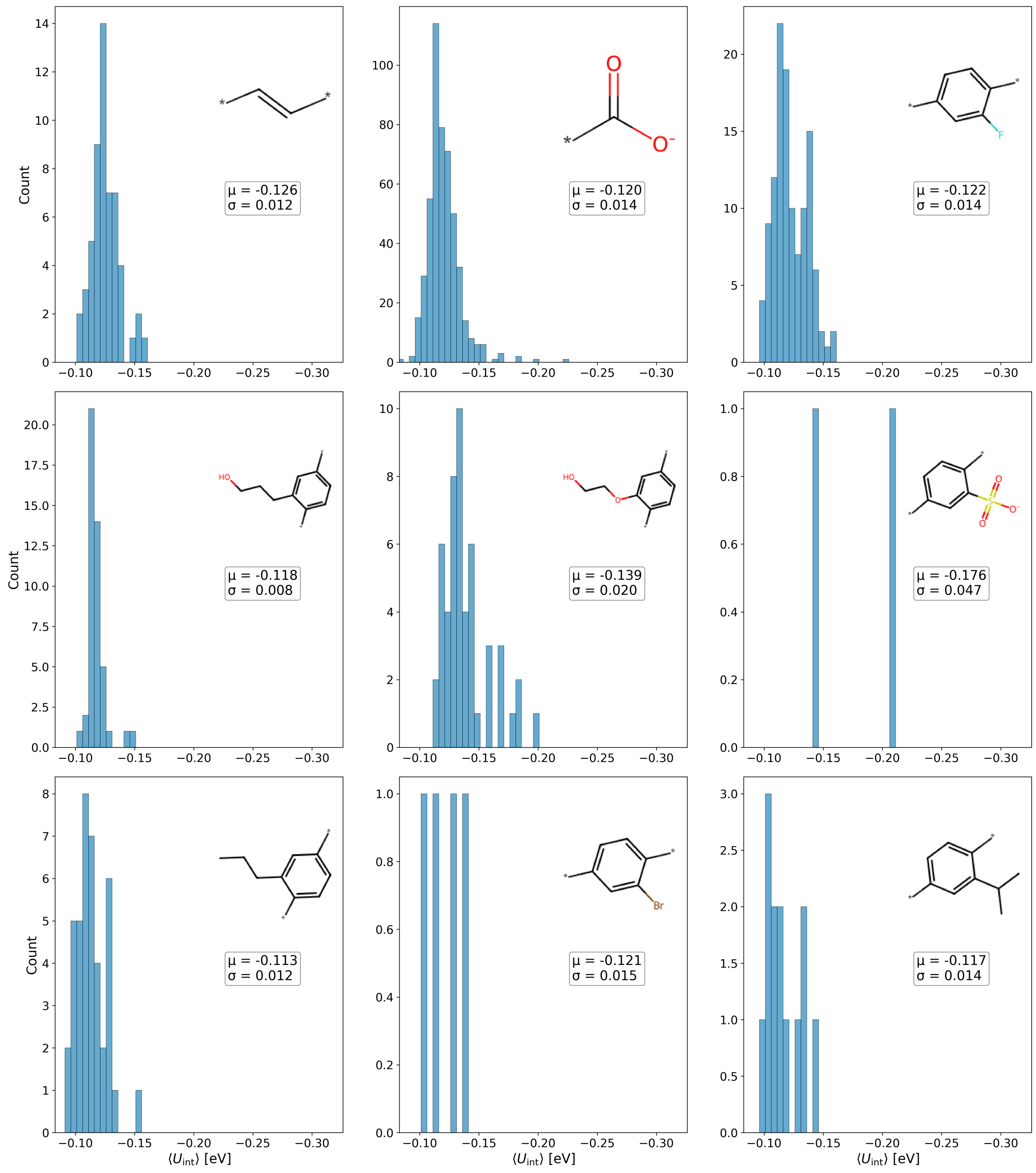

Figure S.18: Average CO_2 interaction energies filtered to CBUs containing each fragment.

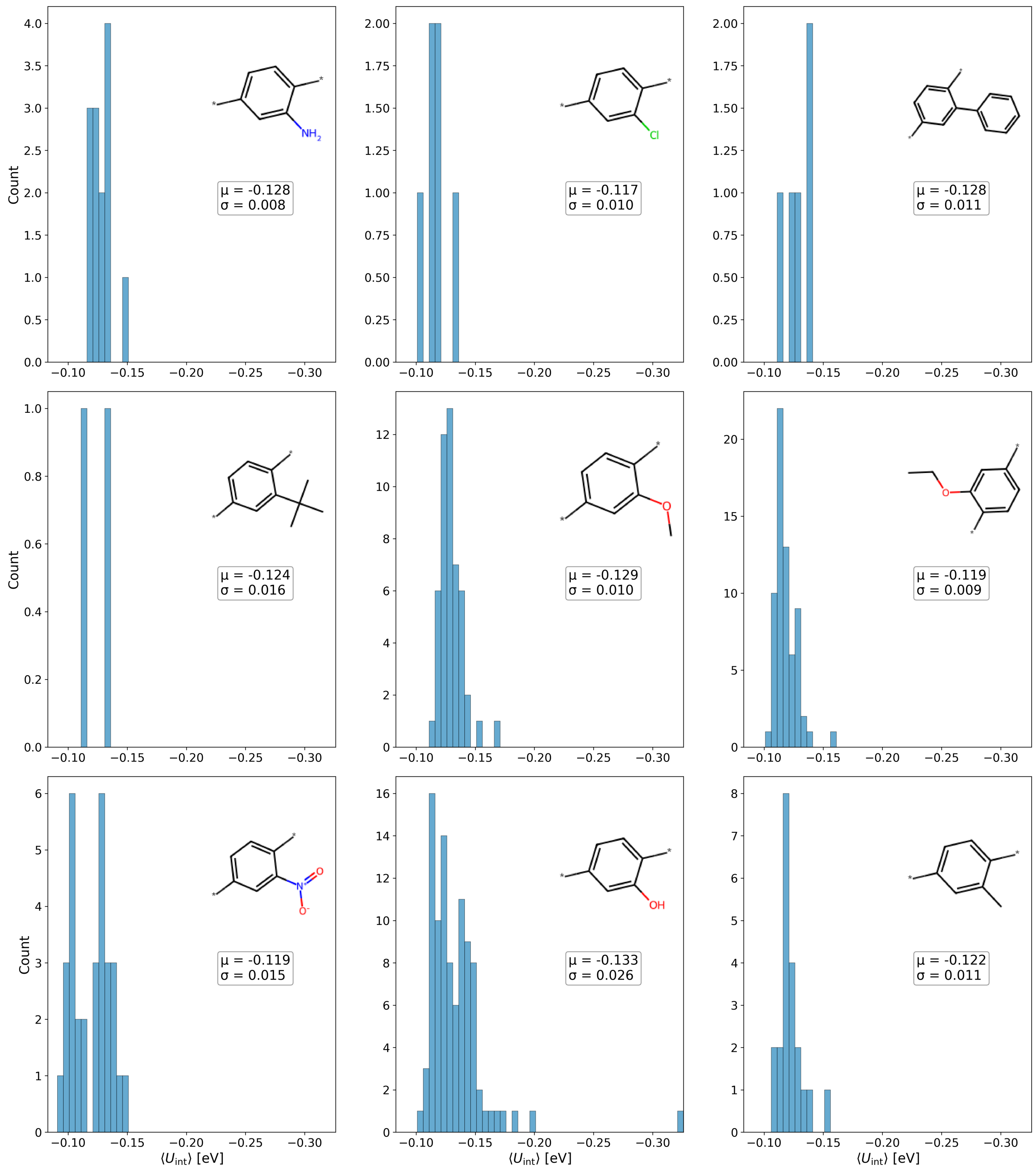

Figure S.19: Average CO_2 interaction energies filtered to CBUs containing each fragment. (cont.)

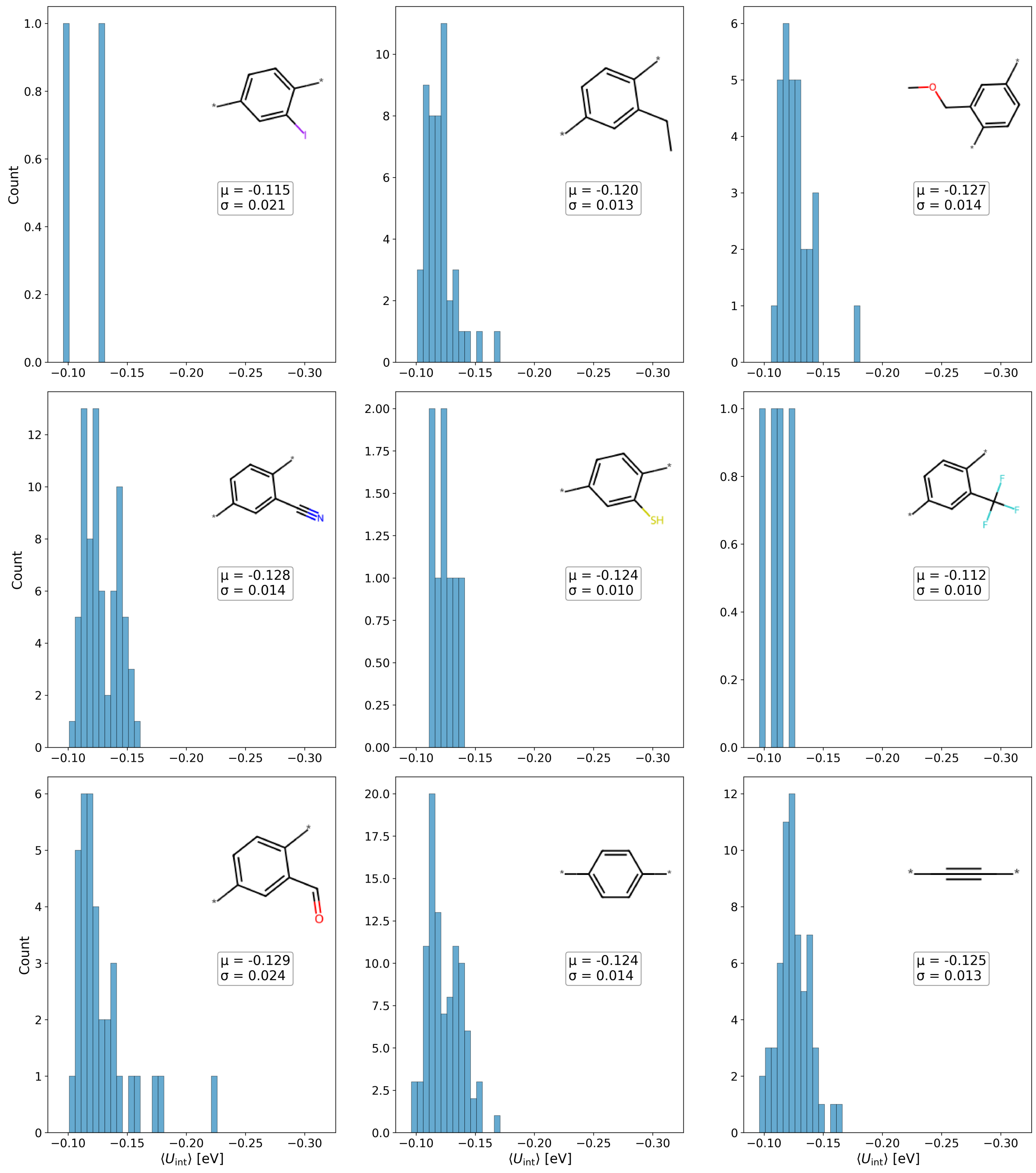

Figure S.20: Average CO_2 interaction energies filtered to CBUs containing each fragment. (cont.)

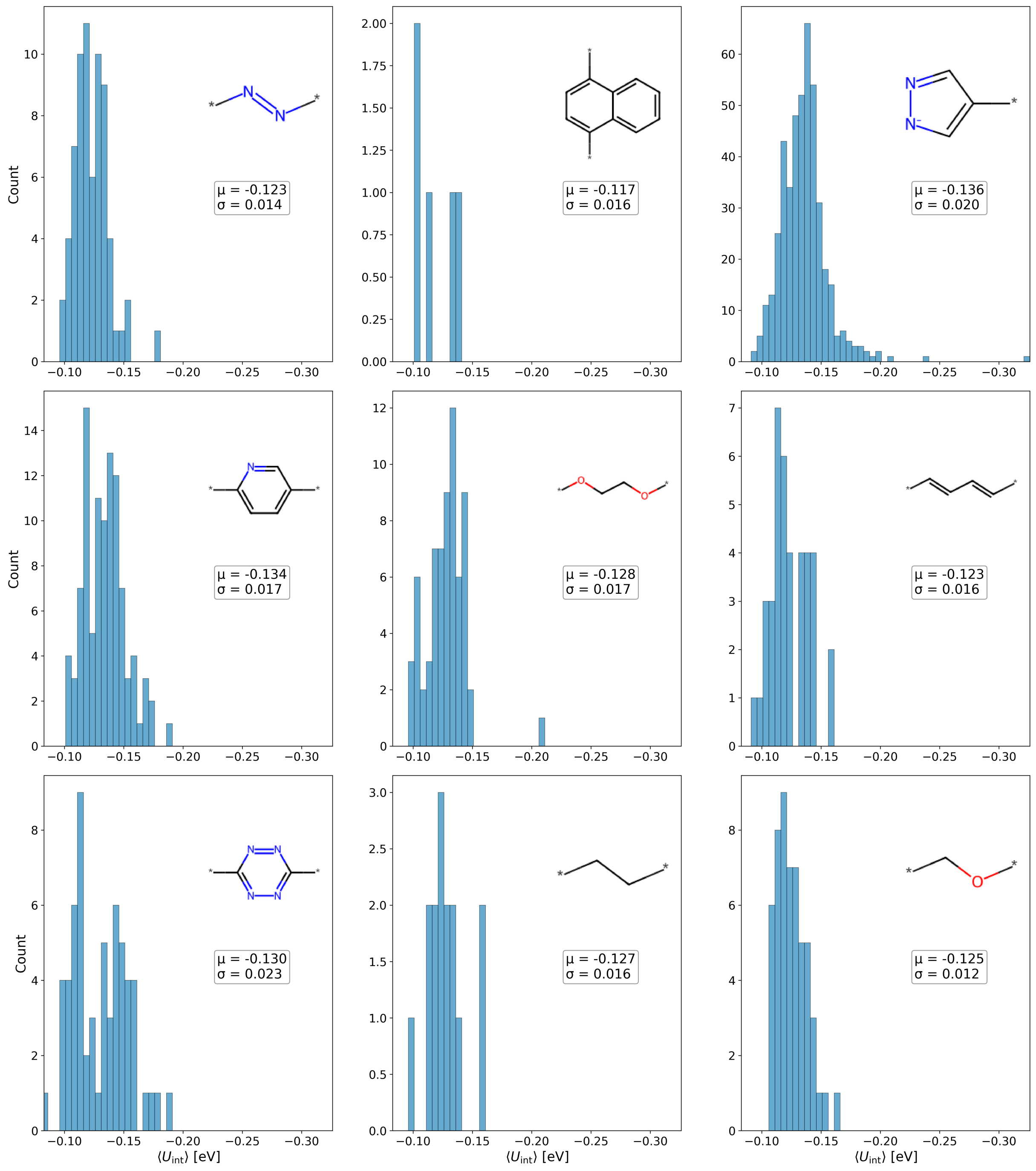

Figure S.21: Average CO_2 interaction energies filtered to CBUs containing each fragment. (cont.)

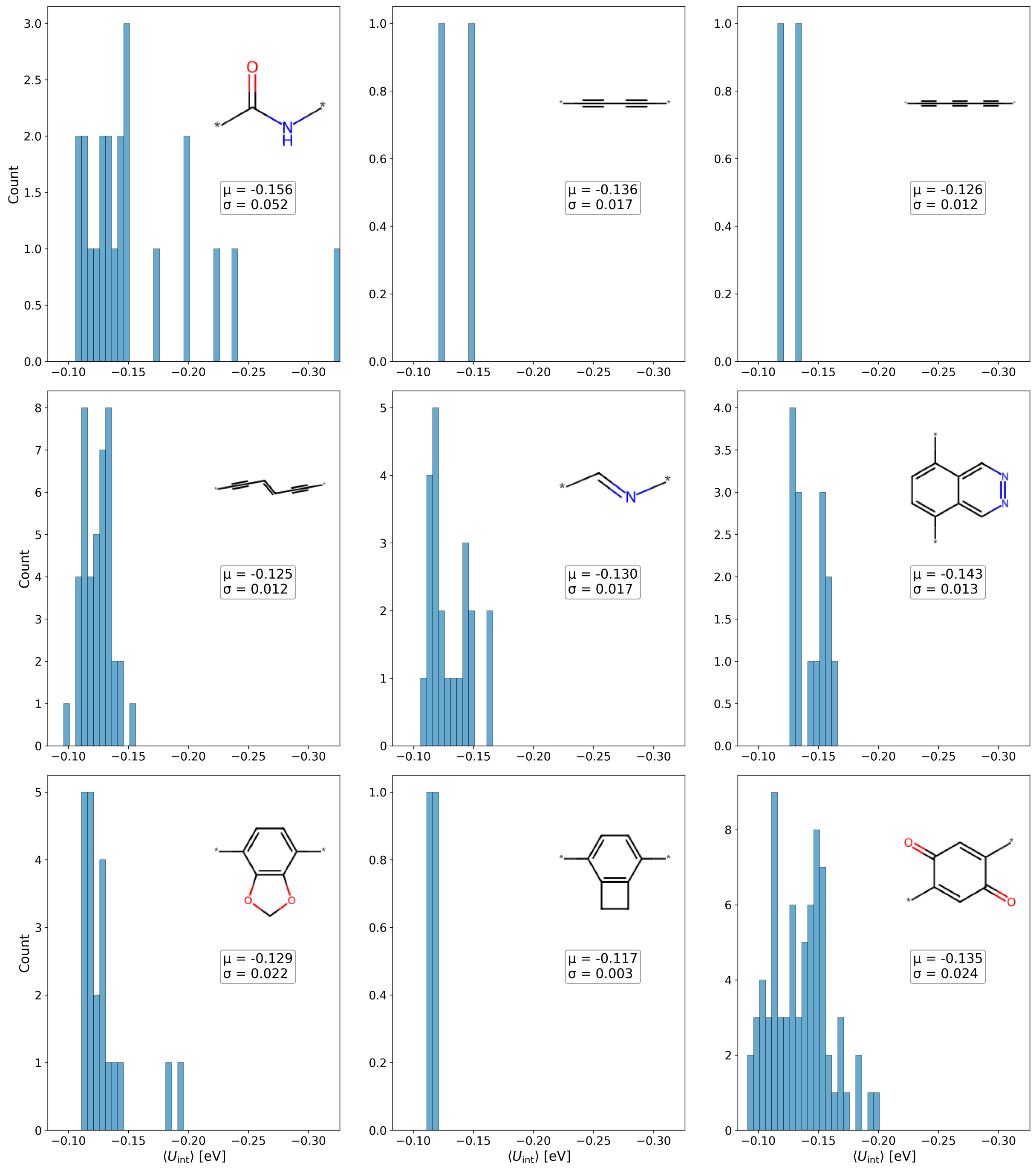

Figure S.22: Average CO_2 interaction energies filtered to CBUs containing each fragment. (cont.)

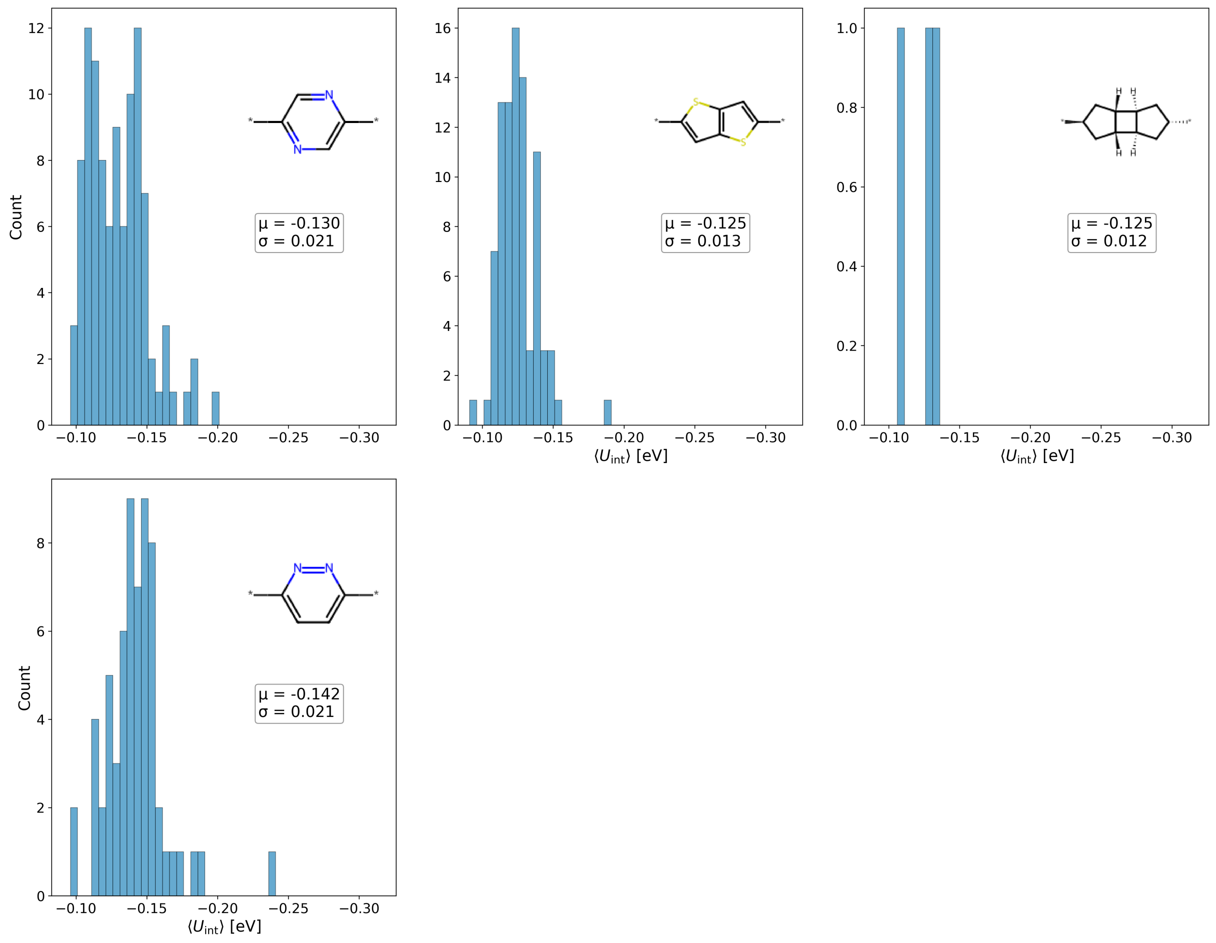

Figure S.23: Average CO_2 interaction energies filtered to CBUs containing each fragment. (cont.)

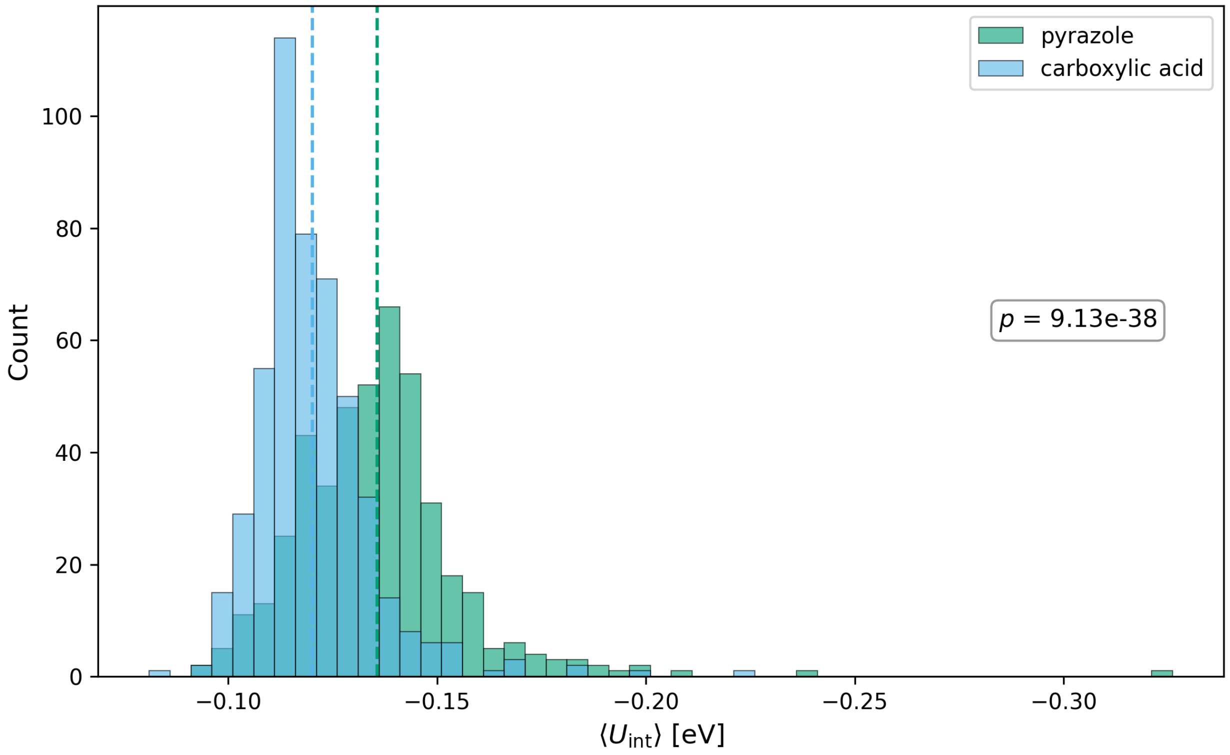

Figure S.24: Average CO_2 interaction energies filtered to each binding group. The means are illustrate by dashed lines and the t-test p-value is annotated.

References

(1) Widom, B. Some Topics in the Theory of Fluids. *The Journal of Chemical Physics* **1963**, *39*, 2808–2812.

(2) Sriram, A.; Brabson, L. M.; Yu, X.; Choi, S.; Abdelmaqsoud, K.; Moubarak, E.; de Haan, P.; Löwe, S.; Brehmer, J.; Kitchin, J. R.; Welling, M.; Zitnick, C. L.; Ulissi, Z.; Medford, A. J.; Sholl, D. S. The Open DAC 2025 Dataset for Sorbent Discovery in Direct Air Capture. 2025; 10.48550/arXiv.2508.03162.

(3) Lim, Y.; Park, H.; Walsh, A.; Kim, J. Accelerating CO2 Direct Air Capture Screening for Metal-Organic Frameworks with a Transferable Machine Learning Force Field. *Matter* **2025**, *8*, 102203.

(4) Wood, B. M. et al. UMA: A Family of Universal Models for Atoms. 2025; 10.48550/arXiv.2506.23971.

(5) Hjorth Larsen, A. et al. The Atomic Simulation Environment—a Python Library for Working with Atoms. *Journal of Physics: Condensed Matter* **2017**, *29*, 273002.

(6) Case, D. H.; Campbell, J. E.; Bygrave, P. J.; Day, G. M. Convergence Properties of Crystal Structure Prediction by Quasi-Random Sampling. *Journal of Chemical Theory and Computation* **2016**, *12*, 910–924.

(7) Gale, J. D. GULP: A Computer Program for the Symmetry-Adapted Simulation of Solids. *Journal of the Chemical Society, Faraday Transactions* **1997**, *93*, 629–637.

(8) Gale, J. D.; Rohl, A. L. The General Utility Lattice Program (GULP). *Molecular Simulation* **2003**, *29*, 291–341.

(9) Addicoat, M. A.; Vankova, N.; Akter, I. F.; Heine, T. Extension of the Universal Force Field to Metal–Organic Frameworks. *Journal of Chemical Theory and Computation* **2014**, *10*, 880–891.

(10) Brandenburg, J. G.; Bannwarth, C.; Hansen, A.; Grimme, S. B97-3c: A Revised Low-Cost Variant of the B97-D Density Functional Method. *The Journal of Chemical Physics* **2018**, *148*, 064104.

(11) Neese, F.; Wennmohs, F.; Becker, U.; Riplinger, C. The ORCA Quantum Chemistry Program Package. *The Journal of Chemical Physics* **2020**, *152*, 224108.
